# Synthesis, Physicochemical Properties, and Biological Activities of 4-(*S*-Methyl-*N*-(2,2,2-Trifluoroacetyl)Sulfilimidoyl) Anthranilic Diamide

**DOI:** 10.3390/molecules24193451

**Published:** 2019-09-23

**Authors:** Hwan Jung Lim, Won Hyung Lee, Seong Jun Park

**Affiliations:** 1 Bio & Drug Discovery Division, Korea Research Institute of Chemical Technology (KRICT), 141 Gajeong-ro, Yuseong-gu, Daejeon 34114, Korea; indium@krict.re.kr; 2 Central Research Institute, Kyung Nong Co. Ltd., 34-14 Summeori-gil, Kyongju 38175, Kyongsangbuk-do, Korea; whlee1@dongoh.co.kr

**Keywords:** anthranilic diamide, sulfilimine, sulfoximine, insecticide

## Abstract

Novel anthranilic diamides with sulfilimidoyl and sulfoximidoyl functionalities were successfully prepared. Among newly-prepared organosulfur compounds, 3-bromo-1-(3-chloropyridin-2-yl)-*N*-(2-methyl-6-(methylcarbamoyl)-4-(methylthio)phenyl)-1*H*-pyrazole-5-carboxamide and (*S*,*E*)-3-bromo-1-(3-chloropyridin-2-yl)-*N*-(2-methyl-4-(S-methyl-*N*-(2,2,2-trifluoroacetyl)sulfinimidoyl)-6-(methylcarbamoyl)phenyl)-1*H*-pyrazole-5-carboxamide showed good levels of efficacy and a strong correlation between insecticidal activities and physical properties, respectively. In particular, available data indicated that the *N*-trifluoroacetyl sulfilimine moiety could be an appealing structural scaffold for the discovery of a new crop-protecting agent.

## 1. Introduction

**Figure 1:**
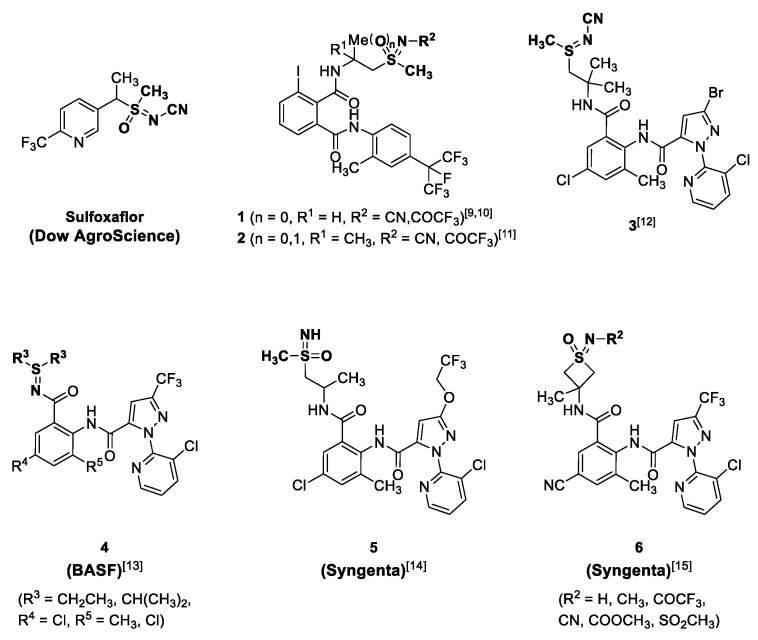
Examples of alkyl sulfilimine- and sulfoximine-substituted insecticides [1,2,3,4,5,6,7,8,9,10,11,12,13,14,15].

After the discovery of sulfoxaflor by Dow AgroScience [1,2,3], compounds with sulfoximine moiety have received remarkable attention in crop protection. Consequently, a large number of studies have examined the chemistry and mode of action of sulfoxaflor and sulfoximine insecticides [4,5,6,7,8]. As shown in Figure 1, the interest in the sulfoximine moiety has led to the discovery of highly-active sulfilimine-containing insecticides **1**–**4** [9,10,11,12,13]. For patent applications, researchers at BASF prepared sulfilimine-based anthranilic diamides **4** and reported that it is highly active in insects which are resistant to ryanodine modulator insecticide [13]. Furthermore, various sulfoximine-containing anthranilamides **6** have been reported by researchers at Syngenta (Figure 1) [14,15]. In addition to crop-protection applications, many research groups reported that sulfoximine could be a bioisostere of sulfones and sulfonamides with enhanced absorption, distribution, metabolism, and excretion (ADME) properties. These suggests that introducing a sulfur–nitrogen bond could be a promising approach for the discovery of new biologically-active molecules [16,17,18,19,20,21,22]. For the preparation of sulfilimine- and sulfoximine-based compounds, synthetic methods and strategies have been widely investigated by many research groups [23,24,25,26,27]. In particular, Bolm et al. reported facile and practical synthetic approaches [28], which are applied in this study.

**Figure 2:**
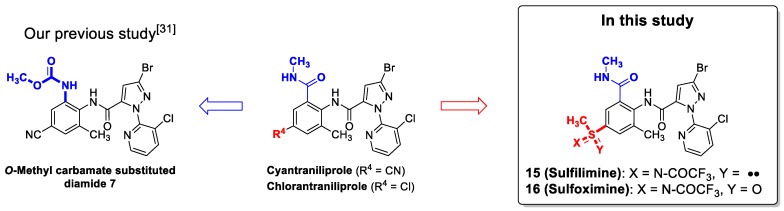
Previously-developed anthranilic diamide [31] and newly-designed insecticides in this study.

Based on previous research and interest in sulfilimine and sulfoximine functionalities, we began to identify these groups for the development of novel insecticides. Because alkyl sulfilimine- and sulfoximine-substituted diamides have so far been reported in the literature (Figure 1), a more focused exploration in our studies was sulfilimine and sulfoximine moieties directly substituted to the 4-position on the anthranilamide ring (Figure 2). We hypothesized that better insecticidal activity could be obtained by these small, lipophilic, electron-withdrawing substituents [1,29,30].

## 2. Results

In our previous study on a novel anthranilic diamide insecticide **7**, we showed that the replacement of *N*-methylcarbamoyl with *O*-methyl carbamate had a beneficial effect, resulting in high insecticidal activity and low toxicity (Figure 2) [31]. Encouraged by these results, we have initially investigated the synthesis of the sulfilimine- and sulfoximine-based diamide **15**, **16** derivatives (Figure 2).

**Scheme 1:**
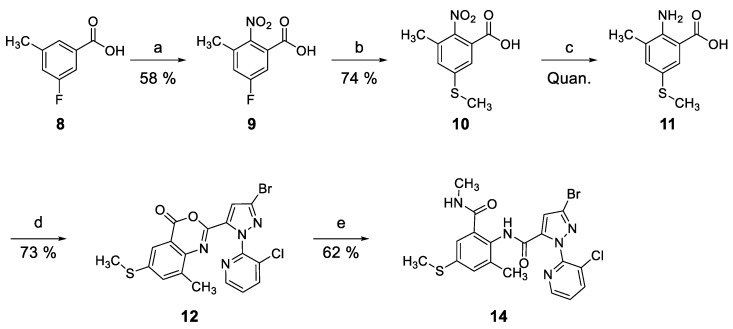
Reagents and conditions: (a) KNO_3_, H_2_SO_4_, RT, 1 h; (b) aqueous NaSMe (21 wt%), 150 °C, 18 h; (c) Na_2_S_2_O_4_, THF/H_2_O (3:2), 60 °C, 0.5 h; (d) 3-bromo-1-(3-chloropyridin-2-yl)-1*H*-pyrazole-5-carboxylic acid **13**, MeSO_2_Cl, pyridine, CH_3_CN, RT, 13 h; (e) CH_3_NH_2_ (2.0 M in THF), THF, 60 °C, 5 h.

As shown in Scheme 1, the targeted compound **14** was successfully prepared using commercially-available 3-fluoro-5-methylbenzoic acid **8**. Regio-selective nitration of **8** provided 2-nitrobenzoic acid **9** in good yield [29,32]. The nitrated product **9** was readily converted to thiomethylated aniline **11** by reaction sequence previously used [31,33,34]. Then, 2-amino-3-methyl-5-(methylthio)benzoic acid **11** was coupled with 3-bromo-1-(3-chloropyridin-2-yl)-1*H*-pyrazole-5-carboxylic acid **13** [12,14,33,34] to give the desired benzoxazinone **12** in good yield [13]. After ring opening reaction of **12** with methylamine [12,14,29,30,34], 4-methylthio anthranilamide **14** was readily obtained.

Next, the sulfur imination and oxidation of diamides **14** was explored (Table 1). Rhodium-catalyzed imination of secondary amide-bearing sulfide **14** and sulfoxide **15b** also provided the desired sulfilimine **15a** and sulfoximine **16a**, respectively (entries 1 and 2, Table 1) [28]. It is worth noting that this metal-catalyzed imination of sulfide **14** led to a mixture of sulfilimine **15a** and sulfoxide **15b** (entry 1, Table 1). For the 4-sulfone group, an oxidation method using MonoPeroxyPhthalate hexahydrate (MMPP·6H_2_O) easily provided the desired compound **16b** (entry 3, Table 1).

For the practical test, newly-prepared compounds **14**–**16** were evaluated for their insecticidal activities against the third instar larvae of *Spodoptera litura* according to the reported leaf-dip method [35].

In addition to sulfilimine **15a** and sulfoximine **16a**, all other synthetic compounds, that is, sulfide **14**, sulfoxide **15b**, and sulfone **16b**, were also tested for their larvicidal activities (Table 2). Among them, sulfide **14** and *N*-trifluoroacetyl sulfilimine **15a** showed good activities with high inhibition of feeding behaviors (eating area—**14** and **15a**: 5–10%, ref.: 0–5%, Table 2) (for images, please see the Supplementary Materials). Highly sensitive and functional group specific insecticidal activities were observed.

**Figure 3:**
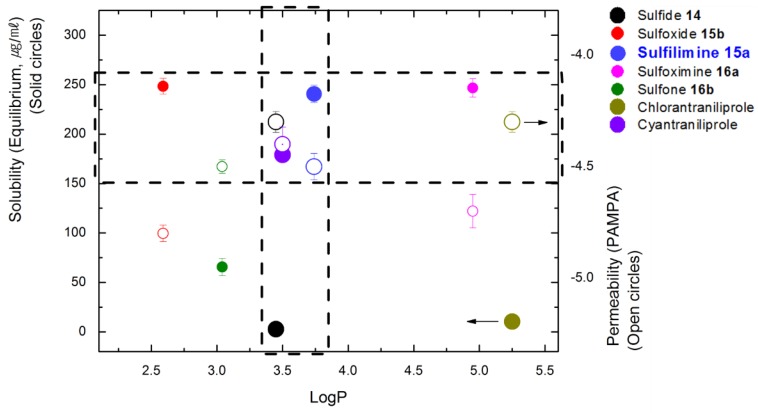
Physical properties of organosulfur-substituted anthranilic diamides **14**–**16** [38,39,40]: Open and solid circles represent permeability (parallel artificial permeability assay, PAMPA) and solubility (equilibrium), respectively; horizontal dash box describes similar permeability (PAMPA) with chlorantraniliprole (dark yellow) and cyantraniliprole (violet); vertical dash box represents similar log P with cyantraniliprole (violet).

Because we believed that the physicochemical properties of newly-prepared diamides play important roles in their insecticidal activities [14,36], the studies were extended to investigate the bioavailability of organosulfur-based crop-protecting agents **14**, **15a**, **15b**, **16a**, and **16b** in terms of plant systemic properties [14] and membrane permeability [36]. As references, their properties of chlorantraniliprole and cyantraniliprole were also measured. Equilibrium solubility, log P, and parallel artificial membrane permeability assay (PAMPA) values are reported in Figure 3 [37].

Although poor water solubility was observed, sulfide **14** displayed relatively low log P and high permeability value (black circle, Figure 3). According to the solubility and log P data, sulfoxide **15b** is believed to have hydrophilic properties (red circle, Figure 3) [41]. In the case of *N*-trifluoroacetyl sulfilimine **15a** and sulfoximine **16a**, it seems that sulfilimine **15a** is more bioavailable than sulfoximine **16a**, especially considering its log P value (blue circle vs pink circle, Figure 3). In physicochemical property tests on organosulfur-based anthranilic diamides, it has been proven that similar values (log P and permeability) of sulfide **14** and *N*-trifluoroacetyl sulfilimine **15a** to cyantraniliprole have resulted in promising insecticidal activities. It is worth noting that sulfilimine **15a** has highly competitive water solubility and permeability to cyantraniliprole, which should aid in plant uptake and translocation [29].

## 3. Material and Methods 

### General Information

Analytical thin layer chromatography (TLC) was performed on Kieselgel 60 F_254_ glass plates precoated with a 0.2 mm thickness of silica gel. The TLC plates were visualized by shortwave (254 nm), potassium permanganate, or ceric ammonium molybdate stain. Flash chromatography was carried out with Kieselgel 60 (230−400 mesh) silica gel. Melting points: Barnstead/Electrothermal 9300, measurements were performed in open glass capillaries. NMR spectra: Bruker AV 300MHz (^1^H-NMR: 300 MHz, ^13^C-NMR: 75 MHz), AV 500MHz (^1^H-NMR: 500 NHz, ^13^C-NMR: 125 MHz), AV2 500MHz (^19^F-NMR: 470 MHz), the spectra were recorded in CDCl_3_ and DMSO-*d*_6_ using TMS as internal standard and are reported in ppm. ^1^H-NMR data are reported as: (s = singlet, d = doublet, t = triplet, q = quartet, br = broad singlet, qui = quintet, oct = octet, m = multiplet; coupling constant(s) in Hz; integration, proton assignment). High resolution mass spectra (HRMS): JEOL JMS-700. All solvents were purified using a column filter solvent purification system before use unless otherwise indicated. Reagents were purchased and used without further purification.

5-Fluoro-3-methyl-2-nitrobenzoic Acid (**9**)

To a solution of 3-fluoro-5-methylbenzoic acid (**8**, 100 mg, 0.6488 mmol) in H_2_SO_4_ (0.8 mL) was added potassium nitrate (72.16 mg, 0.7137 mmol) at 0 °C. After stirring at room temperature for 1 h, the resulting solid was washed with H_2_O to give 5-fluoro-3-methyl-2-nitrobenzoic acid (**9**, 75.3 mg, 58%) as a white solid. Analytical data: lit [33].

3-Methyl-5-(methylthio)-2-nitrobenzoic Acid (**10**)

To a solution of 5-fluoro-3-methyl-2-nitrobenzoic acid (**9**, 387.2 mg, 1.95 mmol) was added an aqueous sodium thiomethoxide (21%, 6.5 mL, 19.5 mmol) at 0 °C. After stirring at 150 °C for 18 h, the reaction mixture was extracted with EtOAc. The organic layer was dried over anhydrous MgSO_4_, filtered and evaporated. The resulting solid was washed with H_2_O to give 3-methyl-5-(methylthio)-2-nitrobenzoic acid (**10**, 325.5 mg, 74%). mp. 178 °C; ^1^H-NMR (300 MHz, DMSO) δ 7.54 (s, 1H), 7.41 (s, 1H), 2.54 (s, 3H), 2.23 (s, 3H); ^13^C-NMR (126 MHz, DMSO) δ 165.4, 147.2, 141.5, 130.2, 129.4, 128.8, 124.4, 16.5, 14.3.

2-Amino-3-methyl-5-(methylthio)benzoic acid (**11**)

To a solution of 3-methyl-5-(methylthio)-2-nitrobenzoic acid (**17**, 42.9 mg, 0.1888 mmol) in THF (3 mL) was added sodium hydrosulfite (75%, 219 mg, 0.9440 mmol) in H_2_O (2 mL) at 0 °C. After stirring at 60 °C for 0.5 h, the reaction mixture was extracted with EtOAc. The organic layer was dried over anhydrous MgSO_4_, filtered, and evaporated. The resulting solid was washed with H_2_O to give 2-amino-3-methyl-5-(methylthio)benzoic acid (**11**, quantitative). mp. 172 °C; ^1^H-NMR (300 MHz, DMSO) δ 7.60 (d, *J* = 2.2 Hz, 1H), 7.23 (d, *J* = 1.6 Hz, 1H), 3.34 (br, 2H), 2.36 (s, 3H), 2.10 (s, 3H); ^13^C-NMR (126 MHz, DMSO) δ 169.4, 148.6, 135.9, 129.8, 124.5, 120.4, 109.7, 17.9, 17.3.

2-(3-Bromo-1-(3-chloropyridin-2-yl)-1*H*-pyrazol-5-yl)-8-methyl-6-(methylthio)-4*H*-benzo[*d*][1,3]oxazin-4-one (**12**)

To a solution of 3-bromo-1-(pyridin-2-yl)-1*H*-pyrazole-5-carboxylic acid (**13**, 432.5 mg, 1.4298 mmol) in dried acetonitrile (1 mL) was added pyridine (0.23 mL, 2.8596 mmol) and methanesulfonyl chloride (0.16 mL, 2.1447 mmol) at 0 °C. After stirring at 0 °C for 30 min, a solution of 2-amino-3-methyl-5-(methylthio)benzoic acid (**11**, 282.67 mg, 1.4298 mmol) in dried acetonitrile and pyridine (0.35 mL, 4.2894 mmol) were added at 0 °C. After stirring at room temperature for 13 h, the reaction mixture was extracted with EtOAc (200 mL). The organic layer was dried over anhydrous MgSO_4_, filtered, and evaporated. The resulting crude residue was purified by column chromatography on silica gel (EtOAc/*n*-Hexane, 1:1) to give 2-(3-bromo-1-(3-chloropyridin-2-yl)-1*H*-pyrazol-5-yl)-8-methyl-6-(methylthio)-4*H*-benzo[*d*][1,3]oxazin-4-one (**12**, 481.4 mg, 73%). ^1^H-NMR (300 MHz, DMSO) δ 8.62 (dd, *J* = 1.4 Hz, *J* = 4.7 Hz, 1H), 8.34 (dd, *J* = 1.4 Hz, *J* = 8.1 Hz, 1H), 7.76 (dd, *J* = 4.7 Hz, *J* = 8.1 Hz, 1H), 7.64 (d, *J* = 2.1 Hz, 1H), 7.57 (d, *J* = 1.5 Hz, 1H), 7.47 (s, 1H), 2.54 (s, 3H), 1.71 (s, 3H); ^13^C-NMR (126 MHz, DMSO) δ 157.7, 148.6, 147.7, 145.5, 140.5, 140.3, 139.9, 136.3, 135.8, 134.3, 128.5, 127.9, 127.3, 120.5, 117.8, 112.5, 15.8, 14.3.

3-Bromo-1-(3-chloropyridin-2-yl)-*N*-(2-methyl-6-(methylcarbamoyl)-4-(methylthio)phenyl)-1*H*-pyrazole-5-carboxamide (**14**)

To a solution of 2-(3-bromo-1-(3-chloropyridin-2-yl)-1*H*-pyrazol-5-yl)-8-methyl-6-(methylthio)-4*H*-benzo[*d*][1,3]oxazin-4-one (**12**, 400 mg, 0.8626 mmol) in THF (1.5 mL) was added methylamine solution (2.0 M in THF, 1.4 mL, 2.8465 mmol) at 0 °C. The mixture was stirred at 0 °C for 5 h, the mixture was extracted with EtOAc (200 mL). The organic layer was dried over anhydrous Na_2_SO_4_, filtered and evaporated. The resulting crude residue was purified by column chromatography on silica gel (CH_2_Cl_2_/MeOH, 20:1) to give 3-bromo-1-(3-chloropyridin-2-yl)-*N*-(2-methyl-6-(methylcarbamoyl)-4-(methylthio)phenyl)-1*H*-pyrazole-5-carboxamide (**14**, 263.4 mg, 62%). mp. 247 °C; LC/MS: R_t_ = 2.82 mins, *m/z* (ES-) = 494 (M + H for C_19_H_17_BrClN_5_O_2_S); IR (KBr): ν 1654, 1633, 1540, 1535, 1459, 1344, 1311, 1024, 958, 878, 861, 801, 794, 761 cm^−1^; ^1^H-NMR (300 MHz, CDCl_3_) δ 10.00 (s, 1H), 8.45 (s, 1H), 7.83 (d, *J* = 8.0 Hz, 1H), 7.36 (s, 1H), 7.17 (s, 1H), 7.05 (s, 2H), 6.26 (s, 1H), 2.92 (d, *J* = 3.1 Hz, 3H), 2.43 (s, 3H), 2.14 (s, 3H); ^13^C-NMR (126 MHz, DMSO) δ 167.0, 155.5, 148.3, 147.0, 139.5, 139.1, 136.8, 136.6, 134.6, 129.3, 128.6, 127.7, 126.7, 126.5, 122.5, 110.4, 26.0, 17.7, 14.5; HRMS (EI) calcd for C_19_H_17_BrClN_5_O_3_S 494.9975, found 494.9949.

3-Bromo-1-(3-chloropyridin-2-yl)-*N*-(2-methyl-4-(S-methyl-*N*-(2,2,2-trifluoroacetyl)sulfinimidoyl)-6-(methylcarbamoyl)phenyl)-1*H*-pyrazole-5-carboxamide (**15a**) and 3-bromo-1-(3-chloropyridin-2-yl)-*N*-(2-methyl-6-(methylcarbamoyl)-4-(methylsulfinyl)phenyl)-1*H*-pyrazole-5-carboxamide (**15b**)

To a solution of 3-bromo-1-(3-chloropyridin-2-yl)-*N*-(2-methyl-6-(methylcarbamoyl)-4-(methylthio)phenyl)-1*H*-pyrazole-5-carboxamide (**14**, 50 mg, 0.1014 mmol) in dried CH_2_Cl_2_ (1 mL) was added trifluoroacetamide (22.92 mg, 0.2028 mmol), magnesium oxide (16.35 mg, 0.4056 mmol), Rhodium(II) acetate (2.2 mg, 0.005 mmol), and iodobenzene diacetate (49 mg, 0.1521 mmol) at 0 °C. After stirring at room temperature for 13 h, the reaction mixture was extracted with CH_2_Cl_2_ (100 mL). The organic layer was dried over anhydrous Na_2_SO_4_, filtered, and evaporated. The resulting crude residue was purified by column chromatography on silica gel (CH_2_Cl_2_/MeOH, 20:1) to give 3-bromo-1-(3-chloropyridin-2-yl)-*N*-(2-methyl-4-(S-methyl-*N*-(2,2,2-trifluoroacetyl)sulfinimidoyl)-6-(methylcarbamoyl)phenyl)-1*H*-pyrazole-5-carboxamide (**15a**, 17.9 mg, 29%) and 3-bromo-1-(3-chloropyridin-2-yl)-*N*-(2-methyl-6-(methylcarbamoyl)-4-(methylsulfinyl)phenyl)-1*H*-pyrazole-5-carboxamide (**15b**, 11.3 mg, 22%). **15a:** mp. 199–200 °C; IR (KBr): ν 1635, 1535, 1465, 1413, 1354, 1302, 1266, 1183, 1145, 962, 801 cm^−1^; ^1^H-NMR (500 MHz, CDCl_3_) δ 10.70 (s, 1H), 8.43 (d, *J* = 4.6 Hz, 1H), 7.85 (d, *J* = 8.0 Hz, 1H), 7.72 (s, 1H), 7.65 (s, 1H), 7.37 (dd, *J* = 4.7 Hz, *J* = 8.0 Hz, 1H), 7.14 (s, 1H), 6.93 (d, *J* = 4.7 Hz, 1H), 2.94 (d, *J* = 4.7 Hz, 3H), 2.86 (s, 3H), 2.24 (s, 3H); ^13^C-NMR (125 MHz, CDCl_3_) δ 166.0, 164.6 (q, CF_3_*C*O, *J* = 33.4 Hz), 155.4, 148.2, 147.0, 139.2, 139.1, 138.2, 136.6, 135.5, 131.2, 130.3, 127.7, 126.8, 126.5, 124.6, 116.8 (q, CF_3_, *J* = 289.8 Hz), 110.8, 32.8, 26.0, 17.9; ^19^F NMR (470 MHz, CDCl_3_) δ -73.28 ppm; HRMS (EI) calcd for C_21_H_17_BrClF_3_N_6_O_3_S 603.9907, found 603.9898. **15b:** mp. 155 °C; IR (KBr): ν 1654, 1636, 1533, 1459, 1355, 1344, 1297, 1241, 1041, 1024, 959, 797 cm^−1^; ^1^H NMR (500 MHz, CDCl_3_) δ 10.64 (s, 1H), 8.46 (dd, *J* = 1.5 Hz, *J* = 4.7 Hz, 1H), 7.85 (dd, *J* = 1.5 Hz, *J* = 8.0 Hz, 1H), 7.71 (d, *J* = 1.7 Hz, 1H), 7.38 (dd, *J* = 4.7 Hz, *J* = 8.0 Hz, 2H), 7.09 (s, 1H), 6.70 (d, *J* = 4.7 Hz, 1H), 2.97 (d, *J* = 4.8 Hz, 3H), 2.67 (s, 3H), 2.27 (s, 3H); ^13^C-NMR (125 MHz, CDCl_3_) δ 168.3, 155.7, 149.0, 146.9, 143.0, 139.0, 138.9, 137.7, 136.8, 130.2, 129.0, 128.3, 128.3, 125.8, 119.6, 111.0, 44.0, 26.9, 19.6; HRMS (EI) calcd for C_19_H_17_BrClN_5_O_3_S 508.9924, found 508.9893.

3-Bromo-1-(3-chloropyridin-2-yl)-*N*-(2-methyl-4-(S-methyl-*N*-(2,2,2-trifluoroacetyl)sulfonimidoyl)-6-(methylcarbamoyl)phenyl)-1*H*-pyrazole-5-carboxamide (**16a**)

To a solution of 3-bromo-1-(3-chloropyridin-2-yl)-*N*-(2-methyl-6-(methylcarbamoyl)-4-(methylsulfinyl)phenyl)-1*H*-pyrazole-5-carboxamide (**15b**, 30 mg, 0.0587 mmol) in dried CH_2_Cl_2_ (1 mL) was added trifluoroacetamide (13.28 mg, 0.1175 mmol), magnesium oxide (9.46 mg, 0.2348 mmol), Rhodium(II) acetate (1.3 mg, 0.003 mmol), and iodobenzene diacetate (28.36 mg, 0.0881 mmol) at 0 °C. After stirring at room temperature for 12 h, the reaction mixture was extracted with CH_2_Cl_2_ (×2). The organic layer was dried over anhydrous Na_2_SO_4_, filtered and evaporated. The resulting crude residue was purified by column chromatography on silica gel (CH_2_Cl_2_/MeOH, 20:1) to give 3-bromo-1-(3-chloropyridin-2-yl)-*N*-(2-methyl-4-(S-methyl-*N*-(2,2,2-trifluoroacetyl)sulfonimidoyl)-6-(methylcarbamoyl)phenyl)-1*H*-pyrazole-5-carboxamide (**16a**, 15.7 mg, 43%). mp. 213.3 °C; IR (KBr): ν 1678, 1640, 1545, 1468, 1411, 1367, 1315, 1239, 1177, 1151, 1111, 994, 963, 838 cm^−1^; ^1^H-NMR (300 MHz, CDCl_3_) δ 10.79 (s, 1H), 8.46 (dd, *J* = 1.5 Hz, *J* = 4.7 Hz, 1H), 7.93 (d, *J* = 2.0 Hz, 1H), 7.87 (dd, *J* = 1.5 Hz, *J* = 8.0 Hz, 1H), 7.81 (s, 1H), 7.39 (dd, *J* = 4.7 Hz, *J* = 8.0 Hz, 1H), 7.09 (s, 1H), 6.50 (s, 1H), 3.41 (s, 3H), 3.01 (d, *J* = 4.9 Hz, 3H), 2.32 (s, 3H); ^13^C-NMR (125 MHz, CDCl_3_) δ 167.5, 164.1 (CF_3_*C*O), 155.5, 148.8, 146.9, 140.5, 139.1, 138.8, 138.4, 133.4, 131.4, 130.1, 129.1, 128.4, 125.9, 123.5, 115.8 (q, CF_3_, *J* = 288.5 Hz), 111.3, 44.2, 27.1, 19.9; ^19^F NMR (471 MHz, CDCl_3_) δ -76.90 (s); HRMS (EI) calcd for C_21_H_17_BrClF_3_N_6_O_4_S 619.9856, found 619.9852.

3-Bromo-1-(3-chloropyridin-2-yl)-*N*-(2-methyl-6-(methylcarbamoyl)-4-(methylsulfonyl)phenyl)-1*H*-pyrazole-5-carboxamide (**16b**)

To a solution of 3-bromo-1-(3-chloropyridin-2-yl)-*N*-(2-methyl-6-(methylcarbamoyl)-4-(methylthio)phenyl)-1*H*-pyrazole-5-carboxamide (**14**, 30 mg, 0.0609 mmol) in MeOH/CH_2_Cl_2_ (1:5, 1 mL) was added magnesium bis(monoperoxyphthalate) hexahydrate (80%, 75.25 mg, 0.1217 mmol) at 0 °C. After stirring at room temperature for 18 h, the reaction mixture was extracted with EtOAc (×2). The organic layer was dried over anhydrous Na_2_SO_4_, filtered and evaporated. The resulting crude residue was purified by column chromatography on silica gel (EtOAc/*n*-Hexane, 3:1) to give 3-bromo-1-(3-chloropyridin-2-yl)-*N*-(2-methyl-6-(methylcarbamoyl)-4-(methylsulfonyl)phenyl)-1*H*-pyrazole-5-carboxamide (**16b**, 29.9 mg, 93%). mp. 122 °C; IR (KBr): ν 1662, 1543, 1464, 1411, 1342, 1307, 1141, 962, 796, 764 cm^−1^; ^1^H-NMR (300 MHz, DMSO) δ 10.54 (br, 1H), 8.50 (dd, *J* = 1.3 Hz, *J* = 4.7 Hz, 1H), 8.17 (dd, *J* = 1.3 Hz, *J* = 8.1 Hz, 1H), 7.91 (s, 1H), 7.82 (s, 1H), 7.61 (dd, *J* = 4.7 Hz, *J* = 8.1 Hz, 1H), 7.41 (s, 1H), 3.22 (s, 3H), 2.69 (d, *J* = 4.5 Hz, 3H), 2.26 (s, 3H); ^13^C-NMR (126 MHz, DMSO) δ 166.2, 155.5, 148.3, 147.2, 139.4, 139.2, 138.5, 137.8, 137.4, 134.6, 130.1, 127.9, 126.9, 126.7, 124.5, 110.9, 43.5, 26.2, 18.1; HRMS (EI) calcd for C_19_H_17_BrClN_5_O_4_S 524.9873, found 524.9844.

## 4. Conclusions

In summary, novel anthranilic diamides, in which organosulfur groups were substituted at the 4-position on the phenyl ring, were prepared and tested for their insecticidal activities and physical properties. For preparation of the target molecules, we expanded reported sulfur imination procedures [28] to amide groups containing sulfide **14**. Our results concerning the relationship between insecticidal activities and physical properties showed that a better bioavailability profile (relatively low log P and high permeability) results in efficacy of sulfide **14** and sulfilimine **15a** (Table 2 and Figure 3). Due to its higher water solubility, *N*-trifluoroacetyl sulfilimine **15a** could be considered the most promising candidate for discovery of a new diamide insecticide. Notably, these studies have demonstrated that changing the substituents on sulfur atoms could lead to compounds with the desired physicochemical property profiles. Among organosulfur groups, *N*-trifluoroacetyl sulfilimine motif brings about desired properties such as solubility, lipophilicity, and permeability.

## Figures and Tables

**Table 1:**
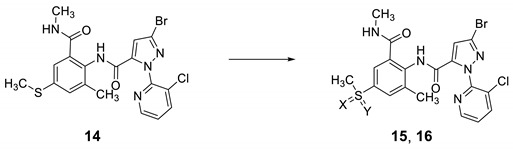
Synthesis of aryl organosulfur analogs of diamide insecticides

Entry	Reaction Conditions ^a^	Substrates	Products			Yield (%) ^b^
		Compd	Compd	X	Y	
1	Imination	**14**	**15a**	N-COCF_3_	••	29
			**15b**	O	••	22
2	Imination	**15b**	**16a**	N-COCF_3_	O	43
3	Oxidation	**14**	**16b**	O	O	93

^a^ Imination: H_2_NCOCF_3_, PhI(OAc)_2_, MgO, Rh_2_(OAc)_2_, CH_2_Cl_2_, 0 °C, 0.5 h; oxidation: MonoPeroxyPhthalate hexahydrate (MMPP·6H_2_O), MeOH/CH_2_Cl_2_ (1:5), RT, overnight, ^b^ after column chromatography; Compd: compound; ••: 1 lone pair on sulfur.

**Table 2:** Insecticidal activities of sulfide- and *N*-trifluoroacetyl sulfilimine-based diamide **14** and **15a** against the third instar larvae of Spodoptera litura.

Entry	Diamides		Against the Third Instar Larvae of *S. litura* ^a^		
	Compd	Functionality	Larvicidal Activity (%) at Time (h)		Eating Area (%)
			72 h	96 h	96 h
1	**14**	sulfide	46.7	73.3	5–10
2	**15a**	*N*-trifluoroacetyl sulfilimine	46.7	53.3	5–10
3	**15b**	sulfoxide	0	0	>30
4	**16a**	*N*-trifluoroacetyl sulfoximine	0	0	>30
5	**16b**	sulfone	0	0	>30

^a^ Concentration of **14** and **15a** was 50 ppm.

## Supplementary Materials

The following are available online at https://www.mdpi.com/1420-3049/24/19/3451/s1, Figure S1: ^1^H, ^13^C, and ^19^F NMR of compounds **9**–**16**, Figure S2: pH-metric log P of compounds **14**–**16**, Table S1: Larvicidal activity depend on time, Table S2: Picture of eating area.
