# Supplementary material for: Synthesis, Physicochemical Properties, and Biological Activities of 4-(S-Methyl-N-(2,2,2-Trifluoroacetyl)Sulfilimidoyl) Anthranilic Diamide
Source: Molecules. 2019 Sep 23;24(19):3451. doi: 10.3390/molecules24193451 (PMC6804157; doi:10.3390/molecules24193451)
Supplement: Supplementary file 1 [file molecules-24-03451-s001.pdf]

Synthesis, Physicochemical Properties, and Biological Activities of 4-(*S*-methyl-*N*-(2,2,2-trifluoroacetyl)sulfilimidoyl) Anthranilic Diamide

Hwan Jung Lim[1], Won Hyung Lee,[2] Seong Jun Park[1,*]

[1]Bio & Drug Discovery Division, Korea Research Institute of Chemical Technology (KRICT), 141 Gajeong-ro, Yuseong-gu, Daejeon 34114, Korea
[2]Central Research Institute, Kyung Nong Co. Ltd., 34-14 Summeori-gil, Kyongju 38175, Kyongsangbuk-do, Korea

Email: sjunpark@krict.re.kr

Supporting information

Figure S1: ^{1}H, ^{13}C and ^{19}F NMR of compound **9-16** 1

Table S1 and S2: Larvicidal activity against Spodoptera (**14** and **15a**)..................... 11

Table S3. Physical properties of organosulfur-substituted anthranilic diamides 15

Figure S2: pH-metric Log P of compounds **14-16** 16

References.. 37

Figure S1: ^{1}H, ^{13}C and ^{19}F NMR of compound **9-16**

^{1}H and ^{13}CNMR of compound **9**

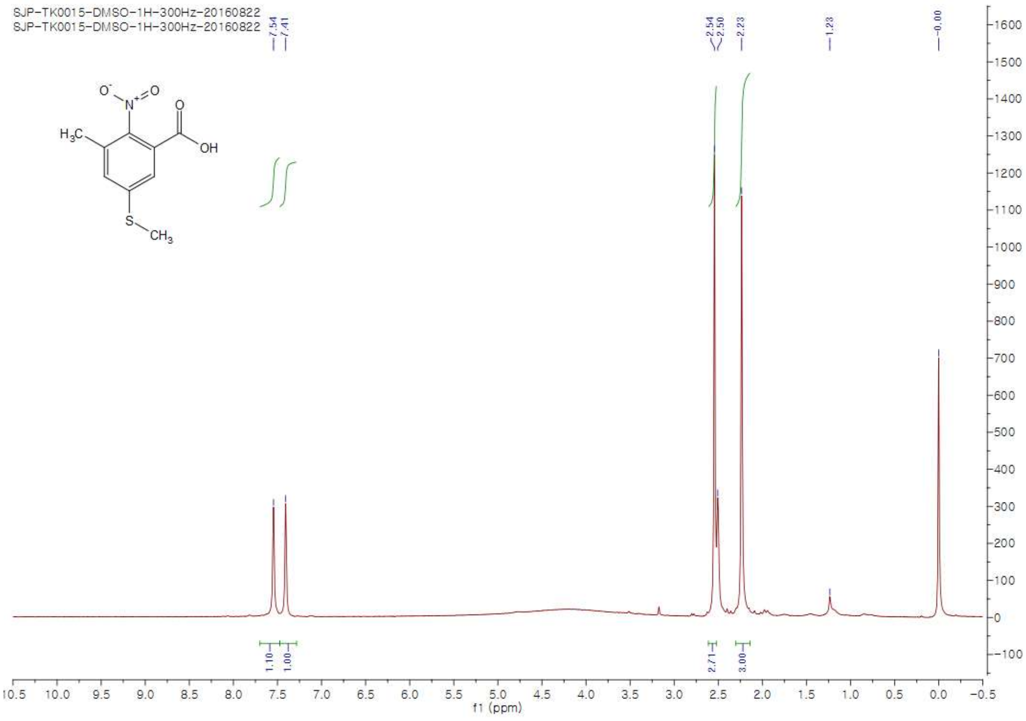

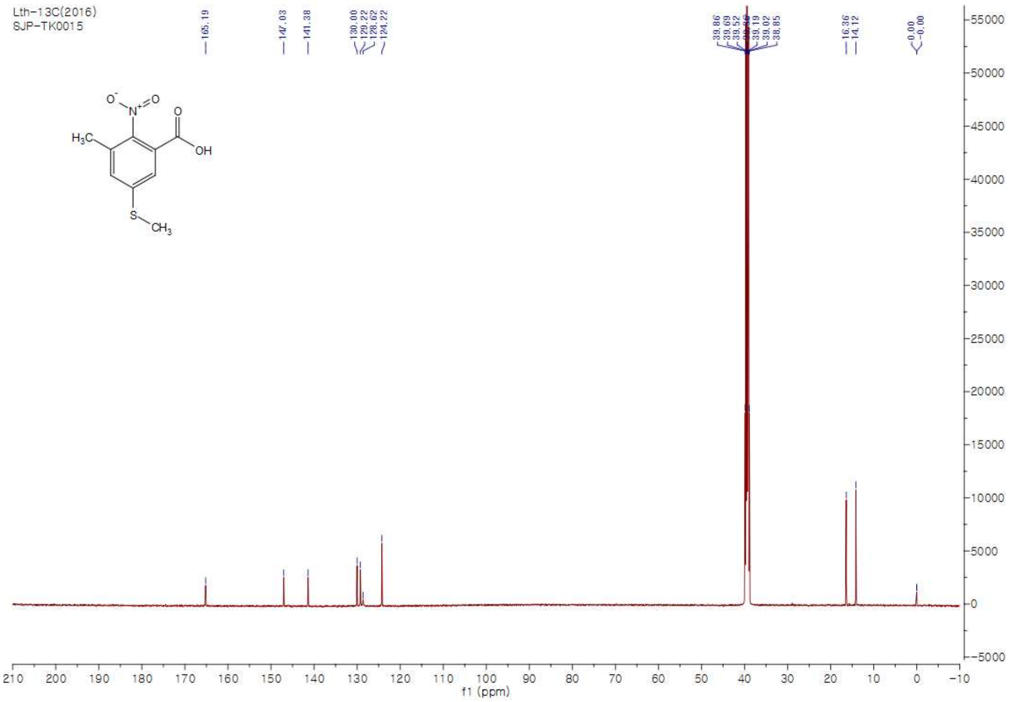

^{1}H and ^{13}C NMR of compound **11**

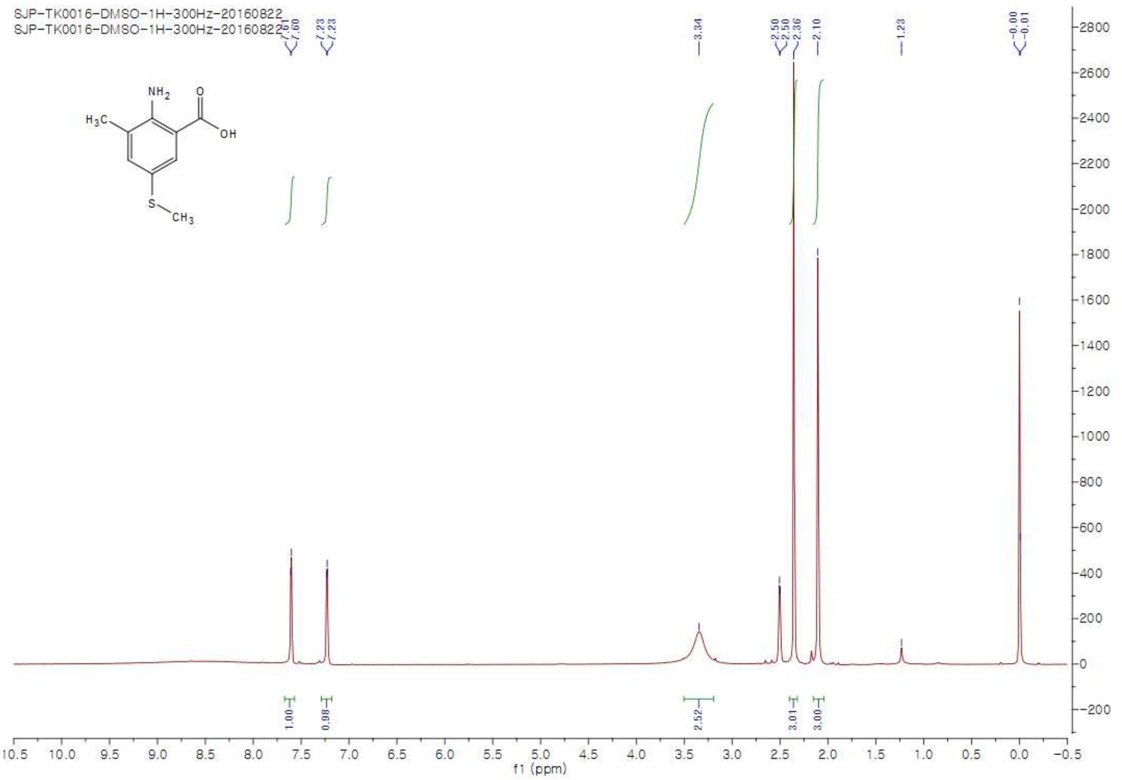

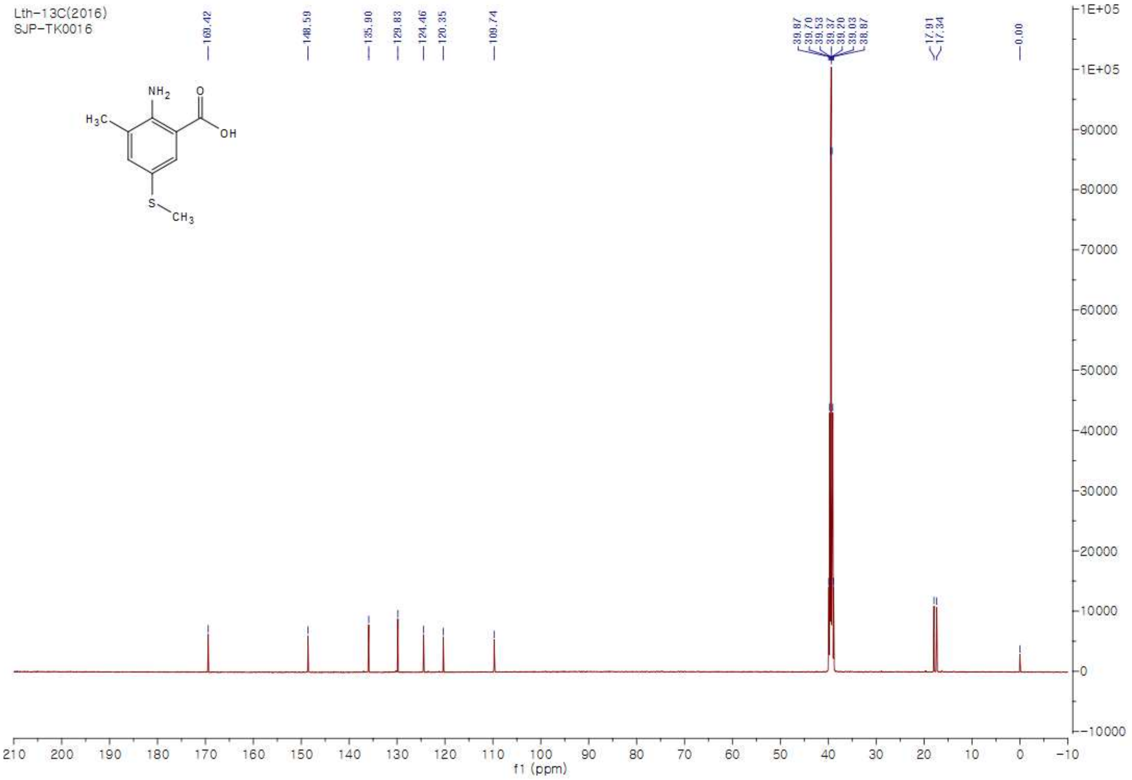

^{1}H and ^{13}C NMR of compound **12**

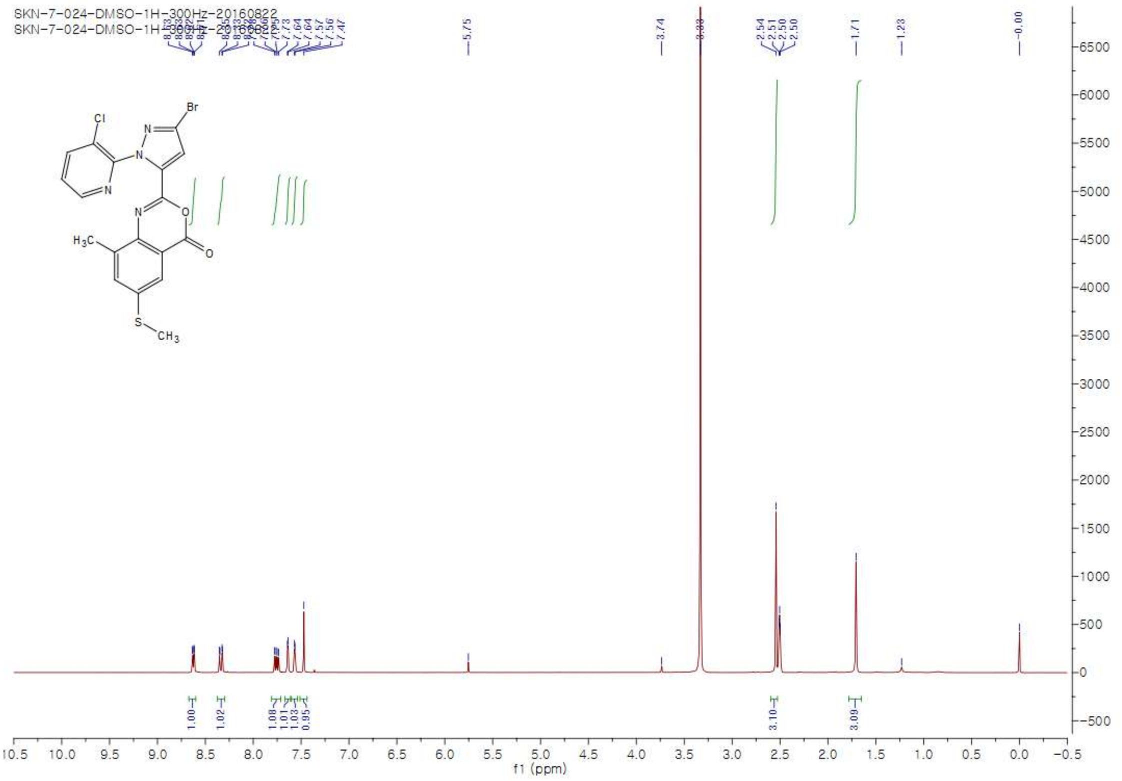

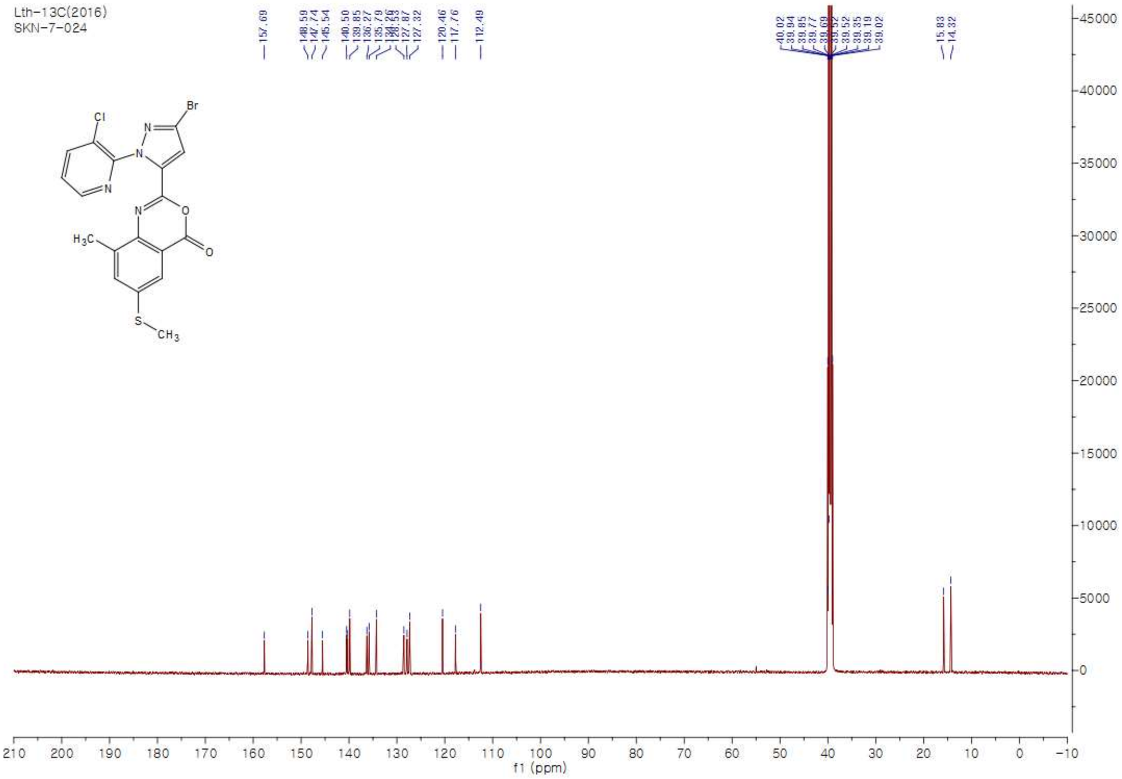

^{1}H and ^{13}C NMR of compound **14**

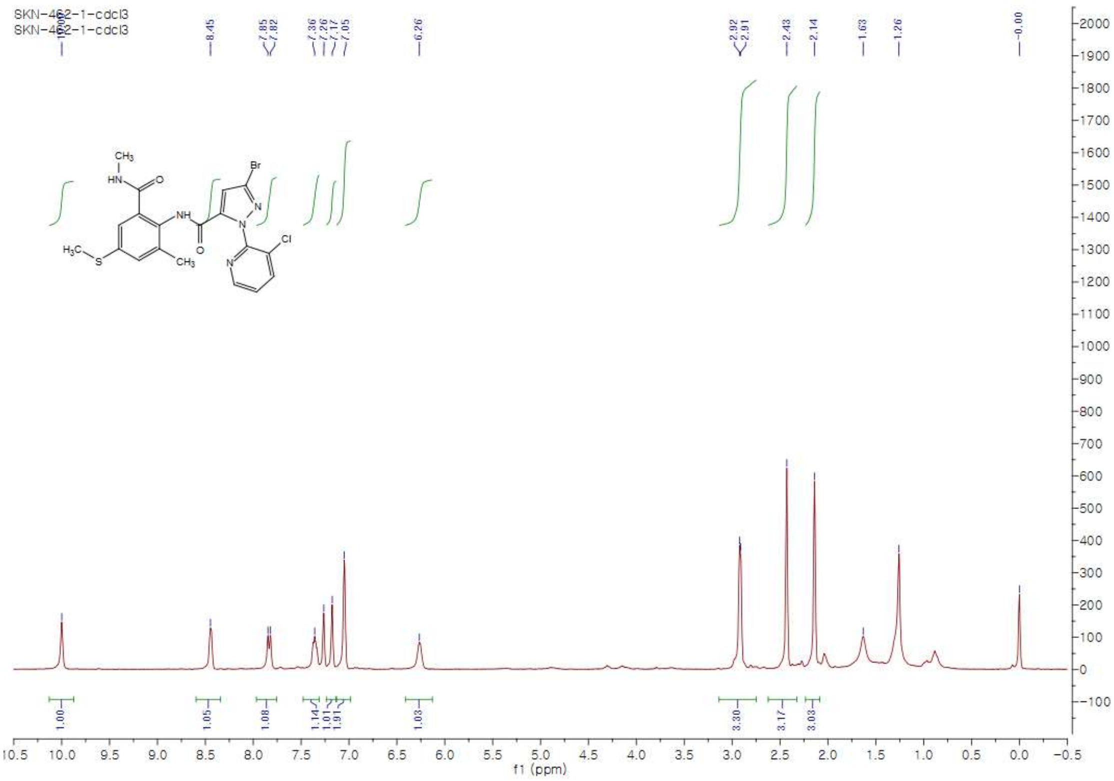

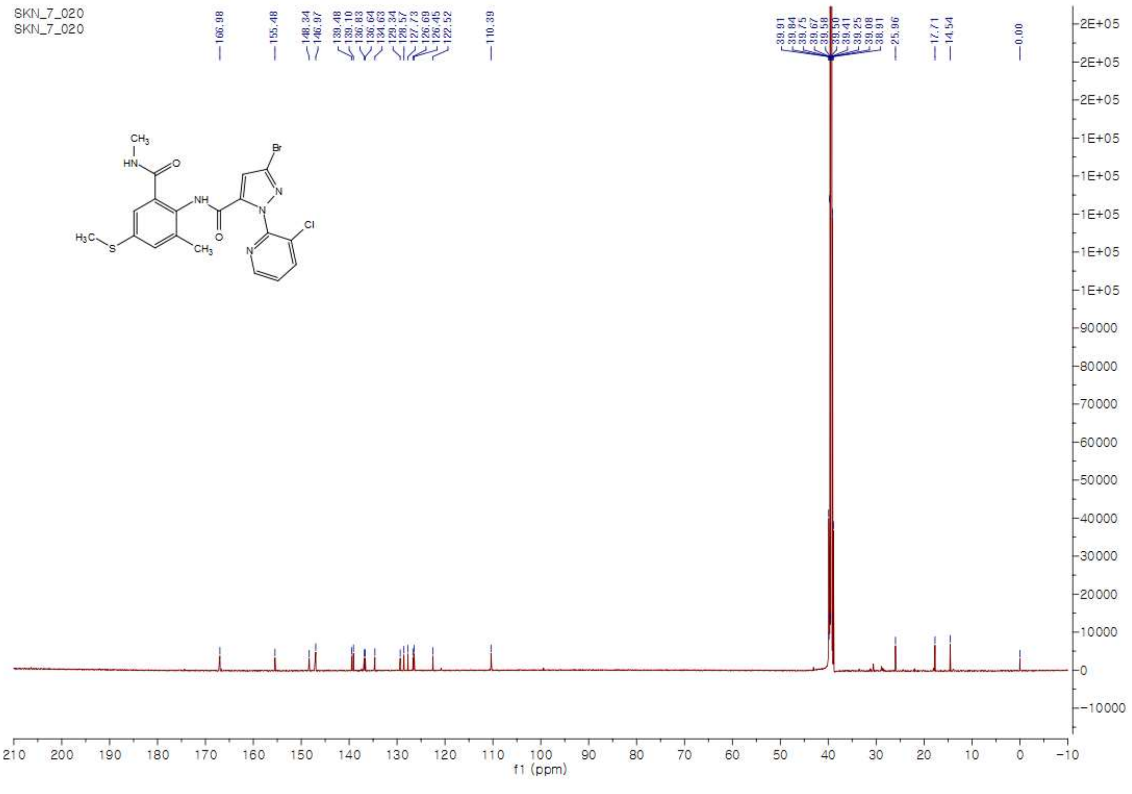

^{1}H and ^{13}C NMR of compound **15a**

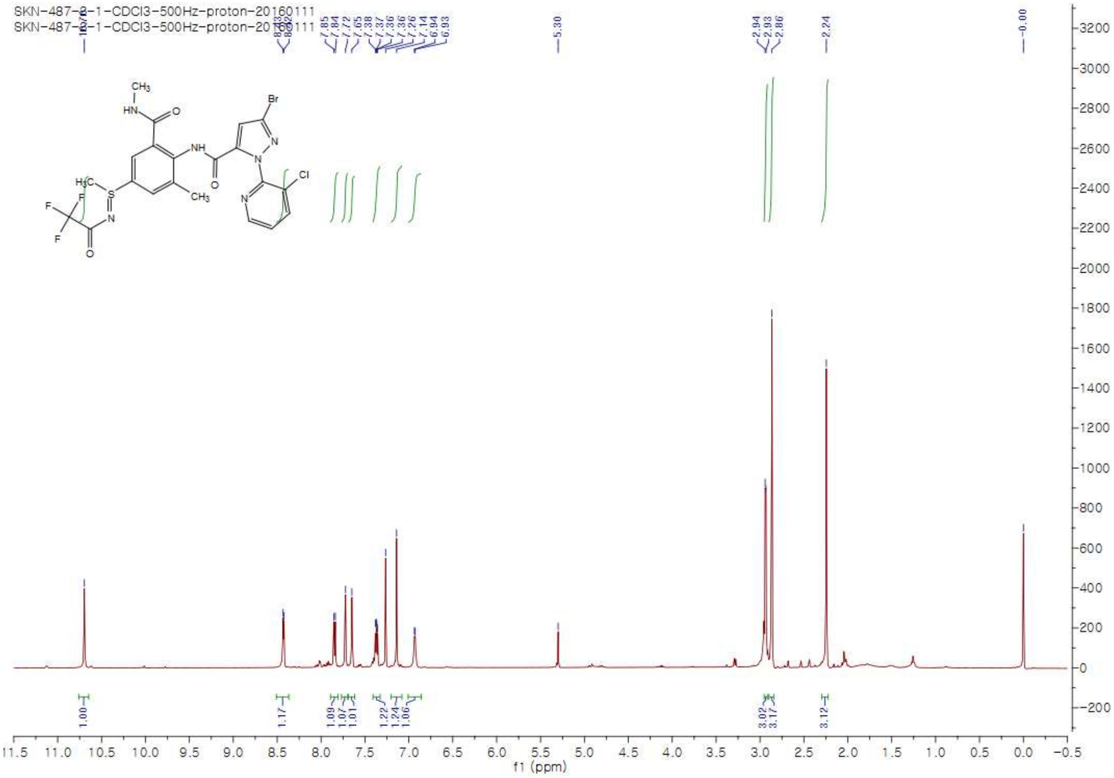

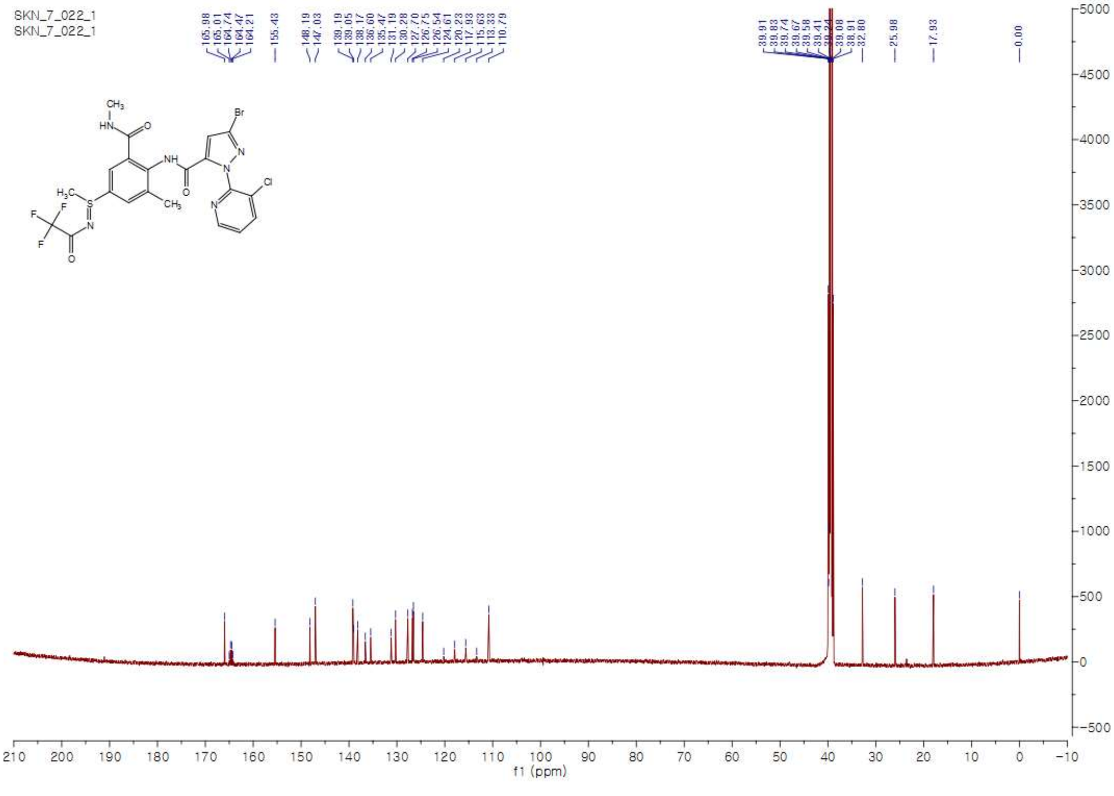

^{19}F NMR of compound **15a**

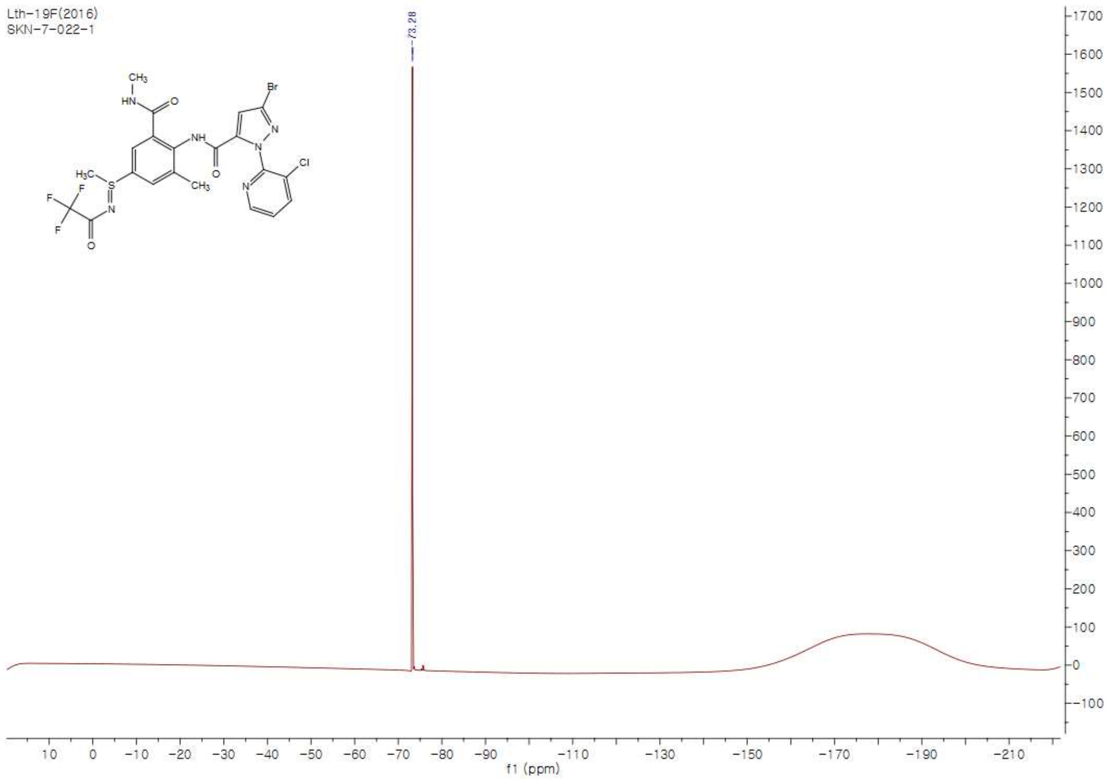

^{1}H and ^{13}C NMR of compound **15b**

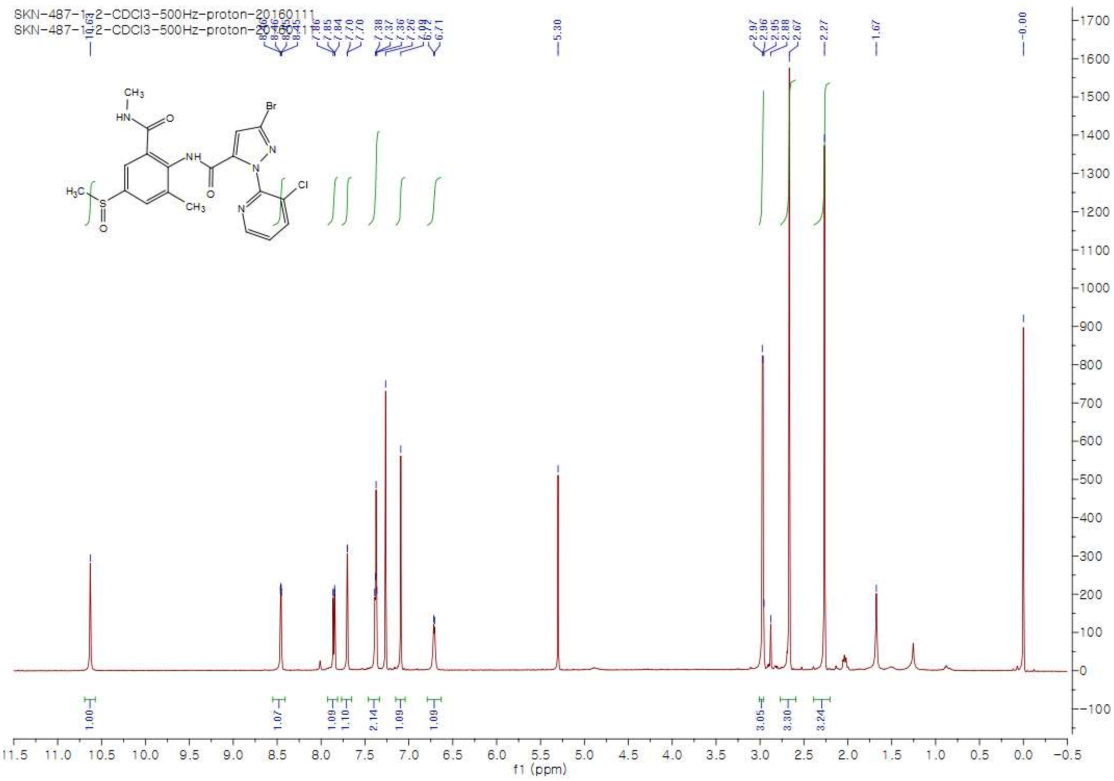

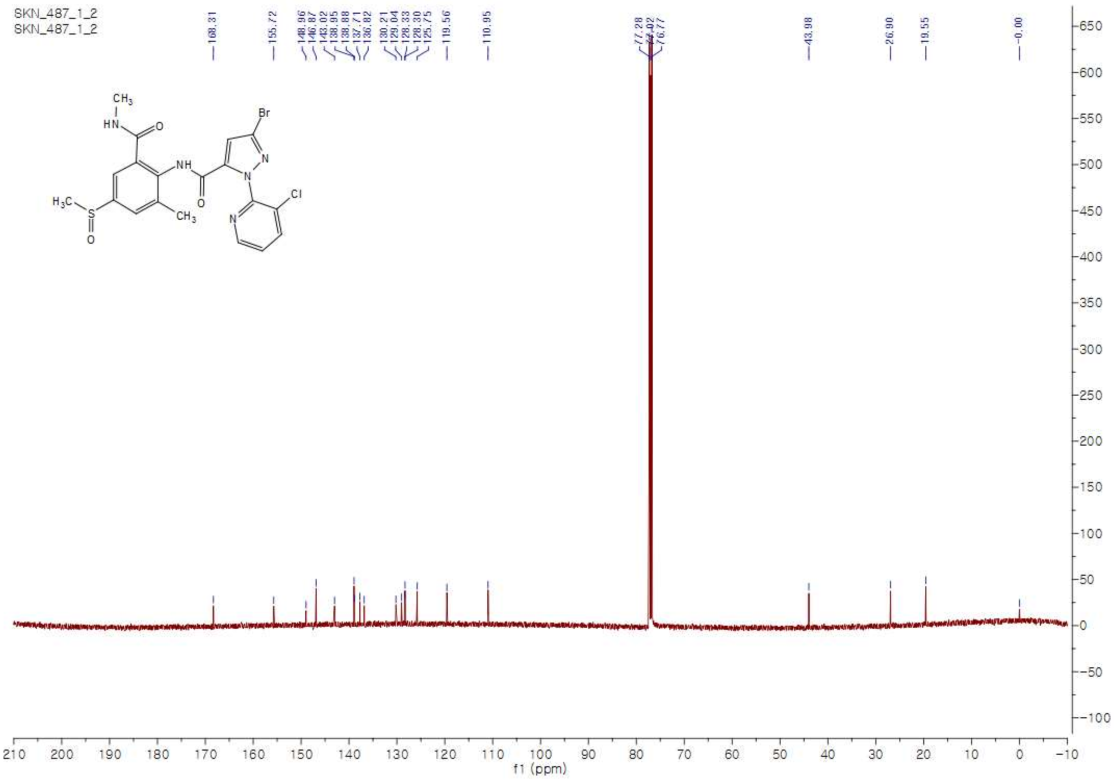

^1H and ^{13}C NMR of compound **16a**

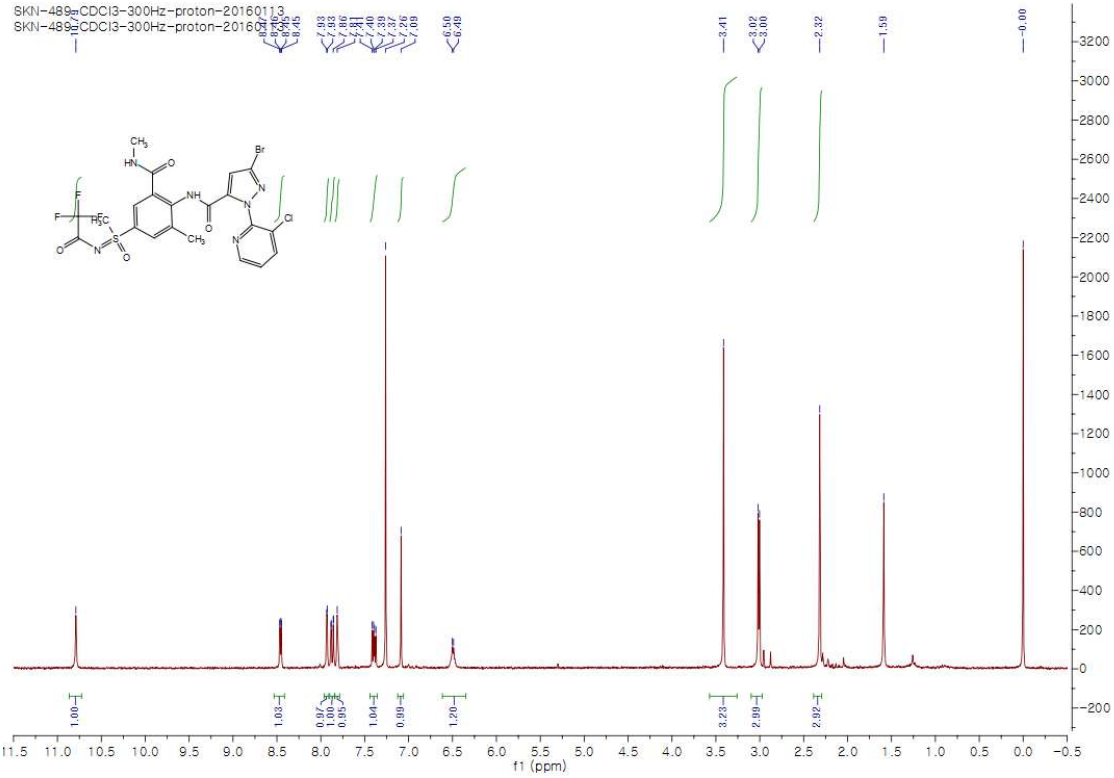

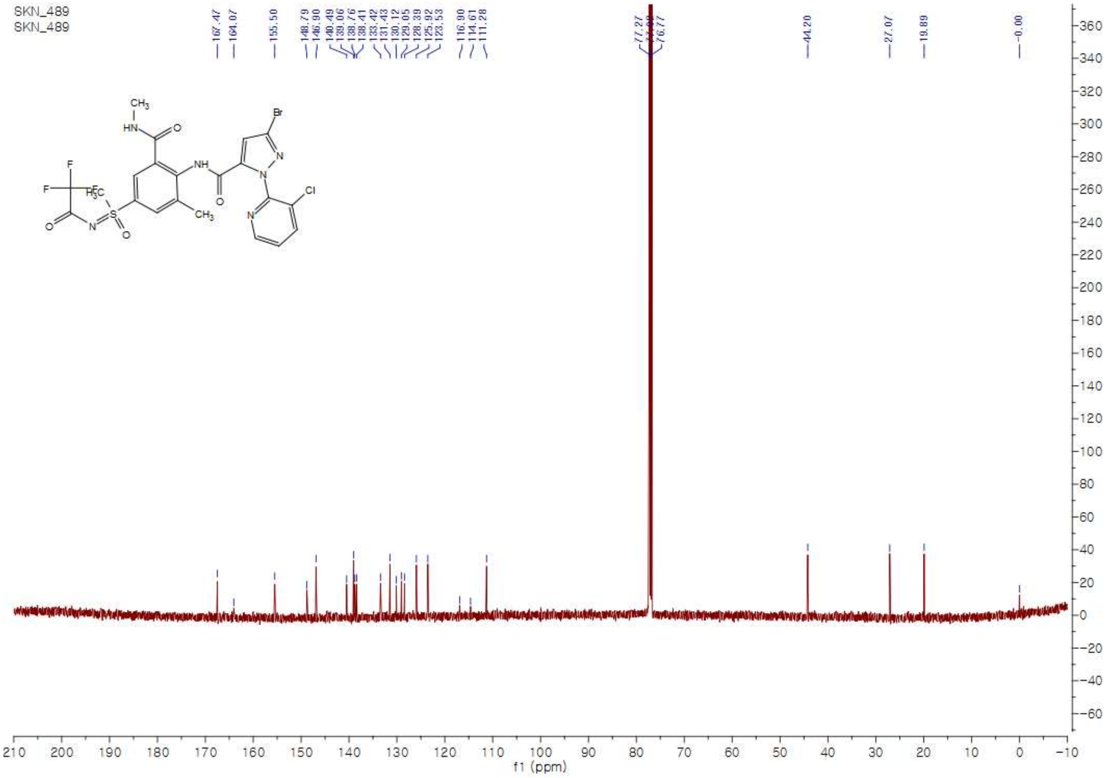

^{19}F NMR of compound **16a**

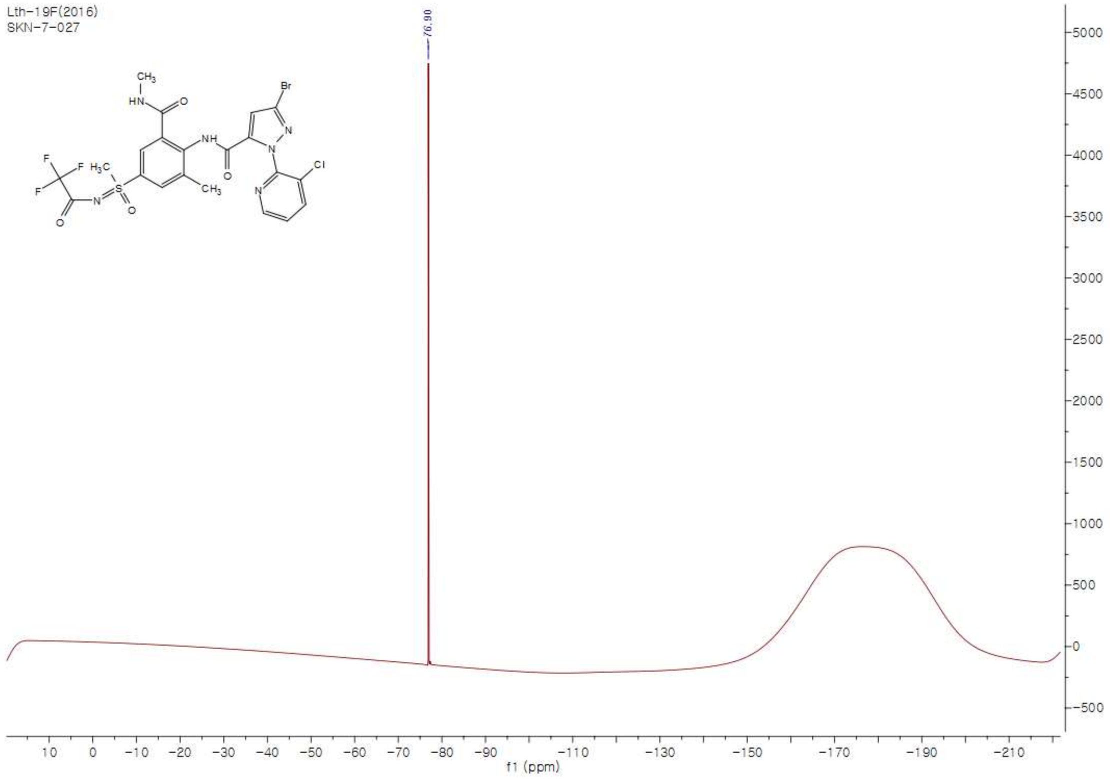

^{1}H and ^{13}C NMR of compound **16b**

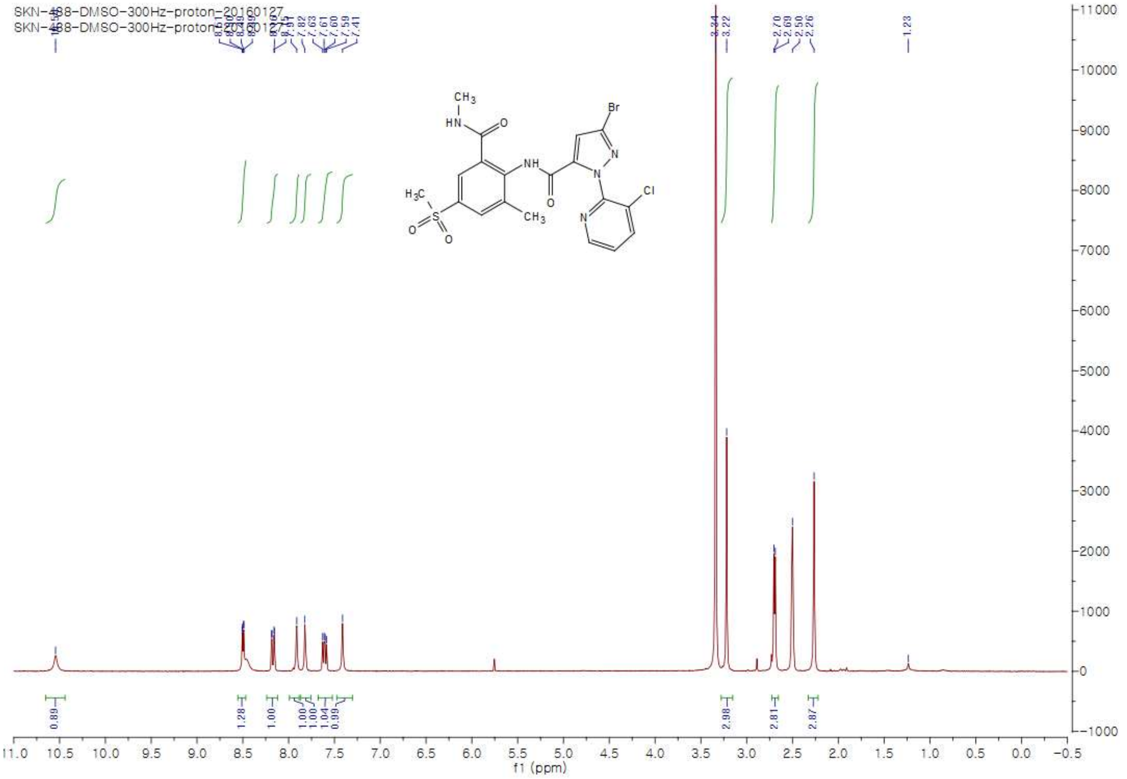

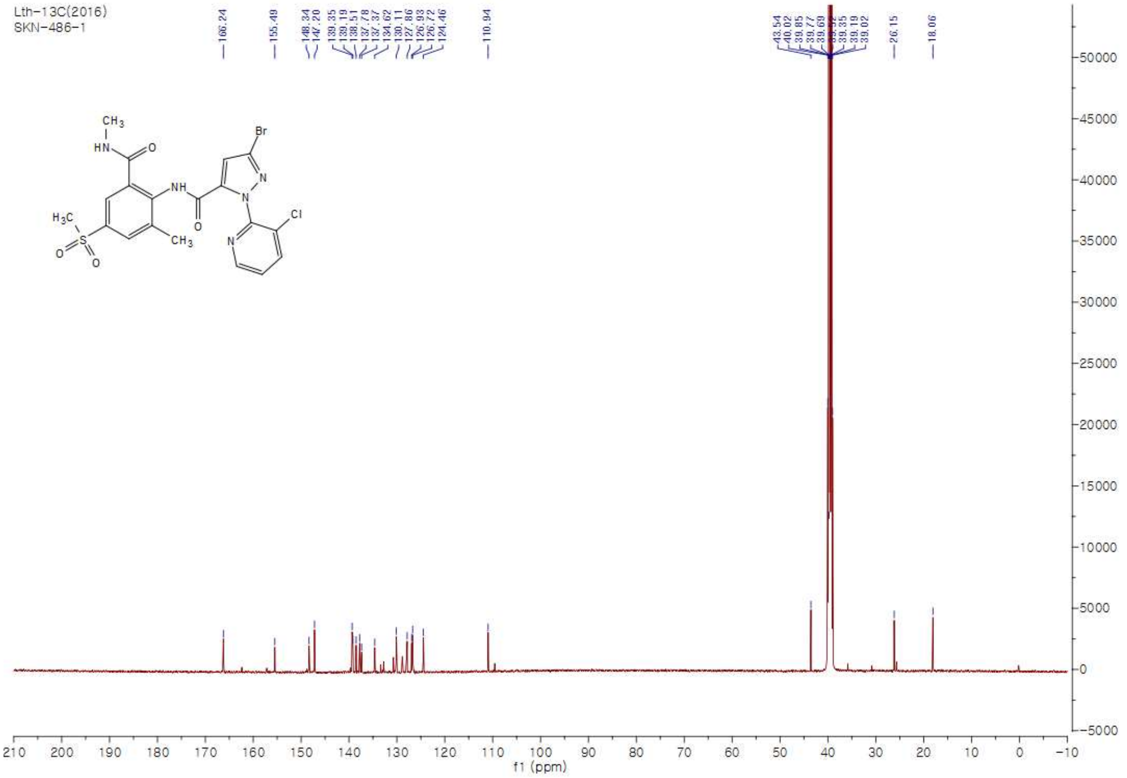

Table S1 and S2: Larvicidal activity against Spodoptera (**14** and **15a**)

The lavicidal activities of synthesized compounds were evaluated by the leaf-dip procedure.[6] The aqueous solution of prepared compounds and chlorantraniliprole in acetone (H_2O : acetone = 95 : 5) were sprayed to a cabbage leave placed on moistened filter paper (disc, diameter 8.8 cm) in petri dishes. After allowing to dry, the dishes were infested with 10 Spodoptera litura (third-instar). After 1, 2, 3, and 4 days, percentage of mortalities was evaluated. The treatments were replicated three times. For negative control, larvicidal activities were 0 % at each time. Ref. is chlorantraniliprole as a positive control.

Table S1. Larvicidal activity depend on time

Entry	Compd	Concentration (ppm)	against the 3rd instar larvae of Spodoptera litura			
			Larvicidal activity (%) at **24 h**			
			1	2	3	Average
1	**14**	50	0	0	0	0
2	Ref.	25	60	30	50	46.7
3	**15a**	50	0	0	0	0
4	Ref.	25	10	20	10	13.3

Entry	Compd	Concentration (ppm)	against the 3rd instar larvae of Spodoptera litura			
			Larvicidal activity (%) at **48 h**			
			1	2	3	Average
1	**14**	50	20	0	20	13.3

2	Ref.	25	90	80	90	86.7
3	**15a**	50	0	0	0	0
4	Ref.	25	50	70	80	66.7

Entry	Compd	Concentration (ppm)	against the 3rd instar larvae of Spodoptera litura			
			Larvicidal activity (%) at **72 h**			
			1	2	3	Average
1	**14**	50	70	30	40	46.7
2	Ref.	25	100	100	100	100
3	**15a**	50	50	40	50	46.7
4	Ref.	25	100	100	100	100

Entry	Compd	Concentration (ppm)	against the 3rd instar larvae of Spodoptera litura			
			Larvicidal activity (%) at **96 h**			
			1	2	3	Average
1	**14**	50	80	70	70	73.3
2	Ref.	25	100	100	100	100
3	**15a**	50	50	50	60	53.3
4	Ref.	25	100	100	100	100

Table S2. Picture of eating area

Entry	Compd	Pictures of eating area_ after 96 h (The 3rd instar stage larvae of Spodoptera litura)
1	**14** (sulfide)	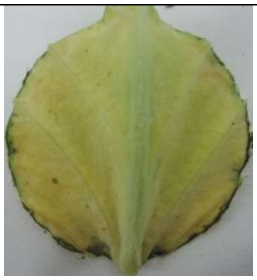 5 - 10 % eating
2	Ref. positive control (chlorantraniliprole)	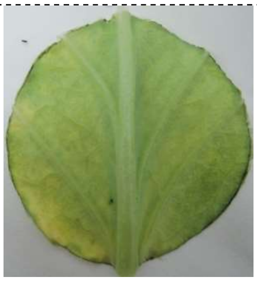 0 – 5 % eating
	Negative control	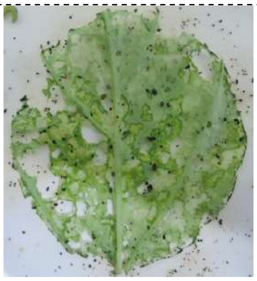

Entry	Compd	Pictures of eating area_ after 96 h (The 3rd instar stage larvae of Spodoptera litura)
2	**15a** (*N*-trifluoroacetyl sulfilimine)	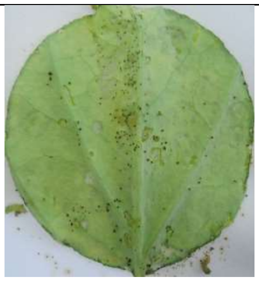 5 - 10 % eating
6	Ref. positive control (chlorantraniliprole)	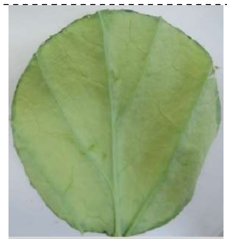 0 – 5 % eating
	Negative control	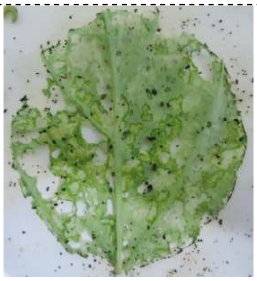

Table S3. Physical properties of organosulfur-substituted anthranilic diamides

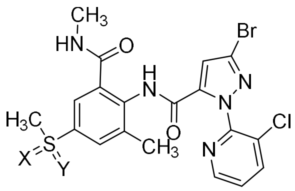

14 -16

Entry	Compd (Functionality)	X	Y	Solubility[a,b] (Equilibrium)	Log P[c,d]	Permeability[e]
1	**14** (Sulfide)	••	••	5.40 ± 0.794 μM (2.67 ± 0.393 μg/mL)	3.45	- 4.3 ± 0.047
2	**15a** (Sulfilimine)	$NCOCF_3$	••	397.3 ± 14.4 μM (240.7 ± 8.737 μg/mL)	3.74	- 4.5 ± 0.059
3	**15b** (Sulfoxide)	O	••	486.1 ± 15.8 μM (248.3 ± 8.083 μg/mL)	2.59	- 4.8 ± 0.037
4	**16a** (Sulfoximine)	$NCOCF_3$	O	396.8 ± 14.9 μM (246.7 ± 9.292 μg/mL)	4.95	- 4.7 ± 0.075
5	**16b** (Sulfone)	O	O	124.5 ± 16.5 μM (65.6 ± 8.693 μg/mL)	3.04	- 4.5 ± 0.030
6	Chlorantraniliprole			21.5 ± 1.59 μM (10.4 ± 0.771 μg/mL)	5.25	- 4.3 ± 0.046
7	Cyantraniliprole			377.9 ± 14.6 μM (179.0 ± 6.928 μg/mL)	3.50	- 4.4 ± 0.077

[a]Method for determination of equilibrium solubility: μSOL[25]; [b]at 25℃ and pH 7.4; [c]Using ACD / Labs T3 method (pH – metric); [d]for graphs, please see the supporting information, [e]Method for determination of permeability: PAMPA

Figure S2: pH-metric Log P of compounds **14-16**

pH-metric Log P of compounds **14**

pH-metric

Sample name:	**SJP-TK0019**	Experiment start time:	**9/1/2016 1:55:54 AM**
Assay name:	**pH-metric medium logP**	Analyst:	
Assay ID:	**16I-01002**	Instrument ID:	**T310022**
Filename:	**D:\data\Customer\16I-01002_SJP-TK0019_pH-metric medium logP.t3r**		

Overall results

RMSD	0.408
Average ionic strength	0.159 M
Average temperature	25.0°C
Partition ratio	0.2745 : 1
Analyte concentration range	2317.8 µM to 2446.0 µM
Total points considered	43 of 56

Warnings and errors

Errors	None
Warnings	Sample concentration factor out of range

Sample

SJP-TK0019 concentration factor	0.456
Acid pKa 1	10.60
logP (neutral XH)	3.45
logP (X -)	2.97

Sample graphs

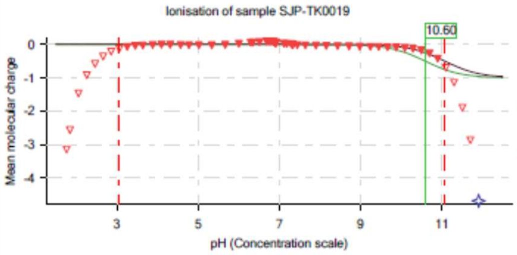

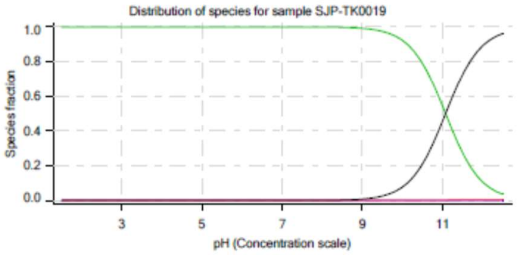

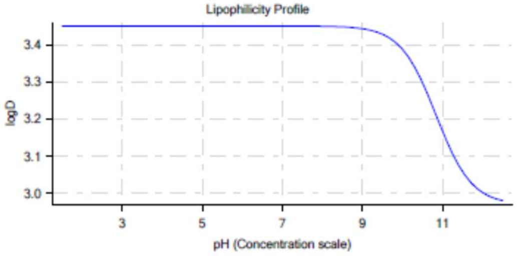

Sample logD and percent species

pH	SJP-TK0019 logD	SJP-TK0019 SJP-TK0019H	SJP-TK0019 SJP-TK0019	SJP-TK0019 SJP-TK0019H*	SJP-TK0019 SJP-TK0019*	Comment
1.000	3.45	0.13 %	0.00 %	99.87 %	0.00 %	
1.200	3.45	0.13 %	0.00 %	99.87 %	0.00 %	Stomach pH
2.000	3.45	0.13 %	0.00 %	99.87 %	0.00 %	
3.000	3.45	0.13 %	0.00 %	99.87 %	0.00 %	
4.000	3.45	0.13 %	0.00 %	99.87 %	0.00 %	
5.000	3.45	0.13 %	0.00 %	99.87 %	0.00 %	

Sample name: **SJP-TK0019**
Assay name: **pH-metric medium logP**
Assay ID: **16I-01002**
Filename: **D:\data\Customer\16I-01002_SJP-TK0019_pH-metric medium logP.t3r**

Experiment start time: **9/1/2016 1:55:54 AM**
Analyst:
Instrument ID: **T310022**

Sample logD and percent species (continued)

pH	SJP-TK0019 logD	SJP-TK0019 SJP-TK0019H	SJP-TK0019 SJP-TK0019	SJP-TK0019 SJP-TK0019H*	SJP-TK0019 SJP-TK0019*	Comment
6.000	3.45	0.13 %	0.00 %	99.87 %	0.00 %	
6.500	3.45	0.13 %	0.00 %	99.87 %	0.00 %	
7.000	3.45	0.13 %	0.00 %	99.86 %	0.01 %	
7.400	3.45	0.13 %	0.00 %	99.85 %	0.02 %	Blood pH
8.000	3.45	0.13 %	0.00 %	99.79 %	0.08 %	
9.000	3.44	0.13 %	0.00 %	99.05 %	0.82 %	
10.000	3.39	0.12 %	0.03 %	92.19 %	7.67 %	
11.000	3.17	0.07 %	0.18 %	54.47 %	45.29 %	
12.000	3.00	0.01 %	0.35 %	10.70 %	88.94 %	

Carbonate and acidity

Carbonate 0.451 mM
Acidity error 0.286 mM

Other graphs

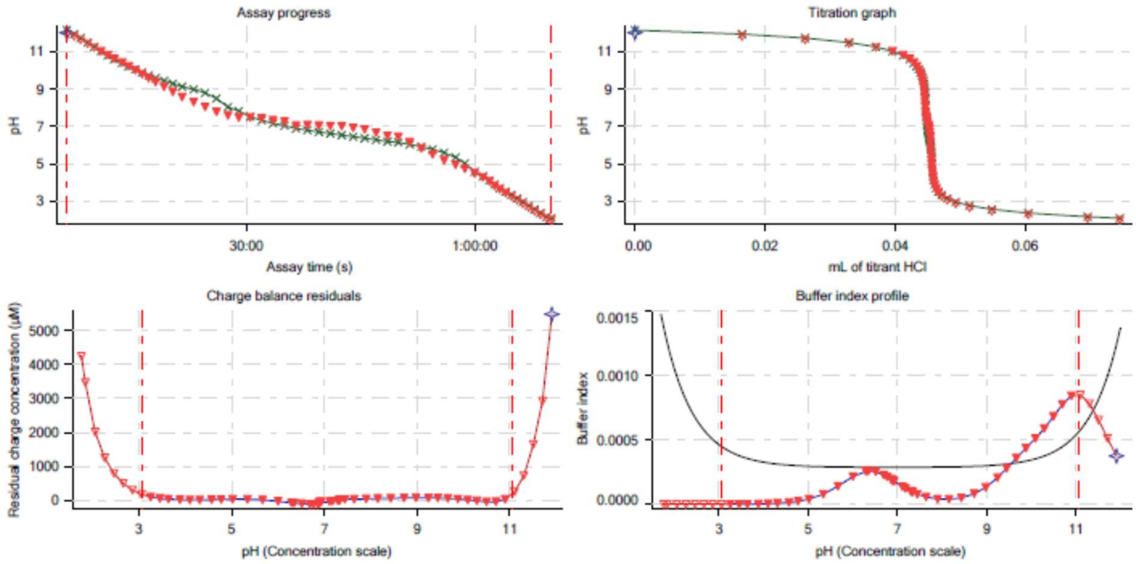

Sample name: **SJP-TK0019**
Assay name: **pH-metric medium logP**
Assay ID: **16I-01002**
Filename: **D:\data\Customer\16I-01002_SJP-TK0019_pH-metric medium logP.t3r**

Experiment start time: **9/1/2016 1:55:54 AM**
Analyst:
Instrument ID: **T310022**

Other graphs (continued)

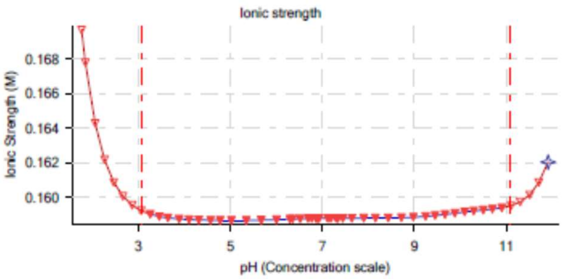

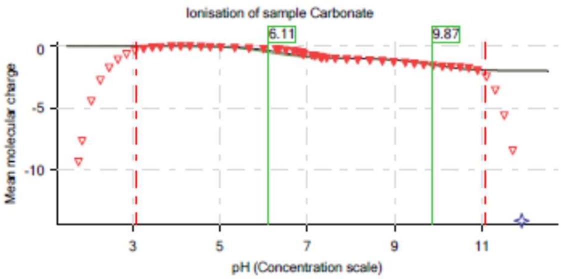

pH-metric Log P of compounds **15b**

pH-metric

Sample name: **SKN-7-022-2**
Assay name: **pH-metric medium logP**
Assay ID: **16I-01008**
Filename: **D:\data\Customer\16I-01008_SKN-7-022-2_pH-metric medium logP.t3r**

Experiment start time: **9/1/2016 9:03:46 AM**
Analyst:
Instrument ID: **T310022**

Overall results

RMSD	0.301
Average ionic strength	0.159 M
Average temperature	25.0°C
Partition ratio	0.2728 : 1
Analyte concentration range	1875.6 µM to 1985.9 µM
Total points considered	43 of 56

Warnings and errors

Errors	None
Warnings	None

Sample

SKN-7-022-2 concentration factor	0.885
Acid pKa 1	10.30
logP (neutral XH)	2.59
logP (X -)	3.25

Sample graphs

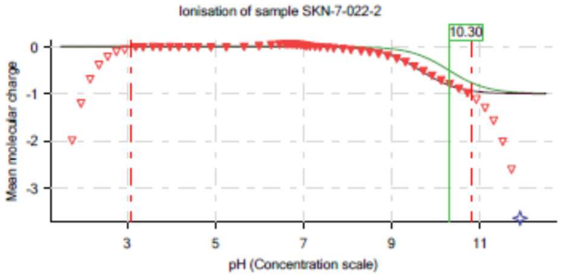

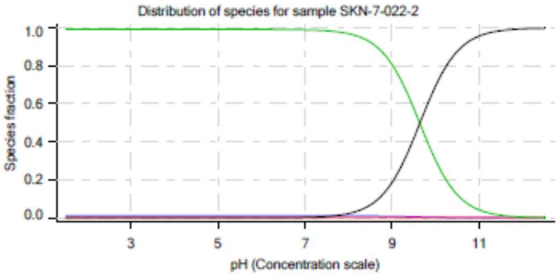

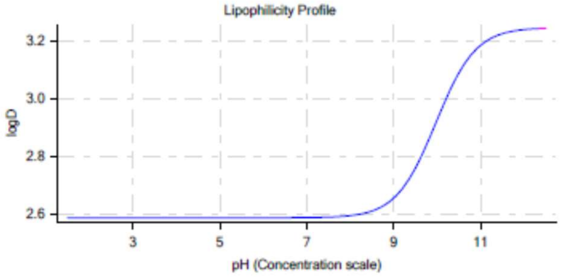

Sample logD and percent species

pH	SKN-7-022-2 logD	SKN-7-022-2 SKN-7-022-2H	SKN-7-022-2 SKN-7-022-2	SKN-7-022-2 SKN-7-022-2H*	SKN-7-022-2 SKN-7-022-2*	Comment
1.000	2.59	0.94 %	0.00 %	99.06 %	0.00 %	
1.200	2.59	0.94 %	0.00 %	99.06 %	0.00 %	Stomach pH
2.000	2.59	0.94 %	0.00 %	99.06 %	0.00 %	
3.000	2.59	0.94 %	0.00 %	99.06 %	0.00 %	
4.000	2.59	0.94 %	0.00 %	99.06 %	0.00 %	
5.000	2.59	0.94 %	0.00 %	99.06 %	0.00 %	

Sample name:	**SKN-7-022-2**	Experiment start time:	**9/1/2016 9:03:46 AM**
Assay name:	**pH-metric medium logP**	Analyst:	
Assay ID:	**16I-01008**	Instrument ID:	**T310022**
Filename:	**D:\data\Customer\16I-01008_SKN-7-022-2_pH-metric medium logP.t3r**		

Sample logD and percent species (continued)

pH	SKN-7-022-2 logD	SKN-7-022-2 SKN-7-022-2H	SKN-7-022-2 SKN-7-022-2	SKN-7-022-2 SKN-7-022-2H*	SKN-7-022-2 SKN-7-022-2*	Comment
6.000	2.59	0.94 %	0.00 %	99.04 %	0.02 %	
6.500	2.59	0.94 %	0.00 %	98.99 %	0.07 %	
7.000	2.59	0.94 %	0.00 %	98.83 %	0.23 %	
7.400	2.59	0.94 %	0.00 %	98.50 %	0.57 %	Blood pH
8.000	2.59	0.92 %	0.00 %	96.86 %	2.22 %	
9.000	2.65	0.77 %	0.04 %	80.73 %	18.47 %	
10.000	2.93	0.29 %	0.14 %	30.29 %	69.28 %	
11.000	3.19	0.04 %	0.20 %	4.18 %	95.58 %	
12.000	3.24	0.00 %	0.21 %	0.43 %	99.35 %	

Carbonate and acidity

- Carbonate 0.440 mM
- Acidity error 0.335 mM

Other graphs

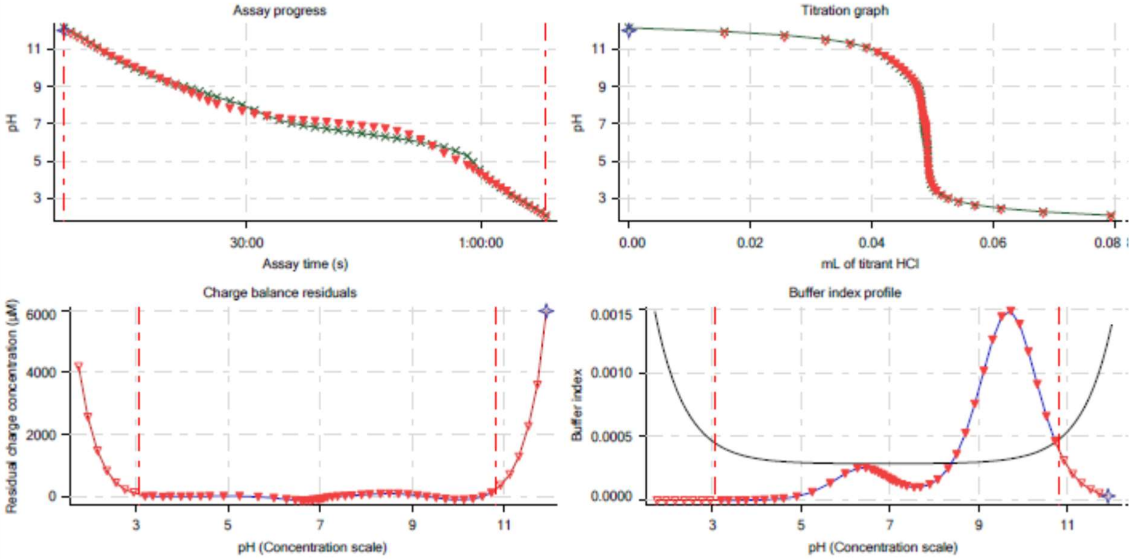

Sample name:	**SKN-7-022-2**	Experiment start time:	**9/1/2016 9:03:46 AM**
Assay name:	**pH-metric medium logP**	Analyst:	
Assay ID:	**16I-01008**	Instrument ID:	**T310022**
Filename:	**D:\data\Customer\16I-01008_SKN-7-022-2_pH-metric medium logP.t3r**		

Other graphs (continued)

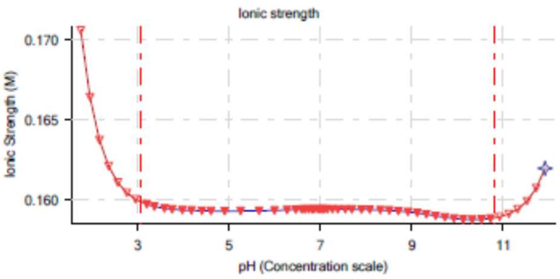

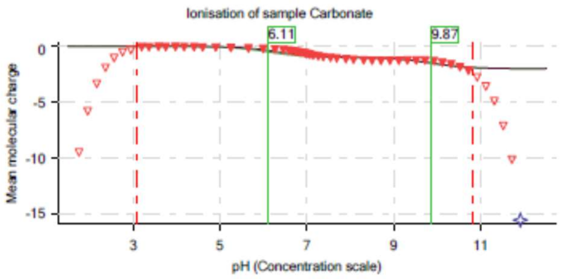

pH-metric Log P of compounds **15a**

pH-metric

Sample name:	**SKN-7-022-1**	Experiment start time:	**9/1/2016 6:47:11 AM**
Assay name:	**pH-metric medium logP**	Analyst:	
Assay ID:	**16I-01006**	Instrument ID:	**T310022**
Filename:	**D:\data\Customer\16I-01006_SKN-7-022-1_pH-metric medium logP.t3r**		

Overall results

RMSD	0.252
Average ionic strength	0.159 M
Average temperature	25.0°C
Partition ratio	0.2740 : 1
Analyte concentration range	1598.6 µM to 1689.8 µM
Total points considered	38 of 54

Warnings and errors

Errors	None
Warnings	Sample concentration factor out of range

Sample

SKN-7-022-1 concentration factor	0.635
Acid pKa 1	10.60
logP (neutral XH)	3.74
logP (X -)	5.35

Sample graphs

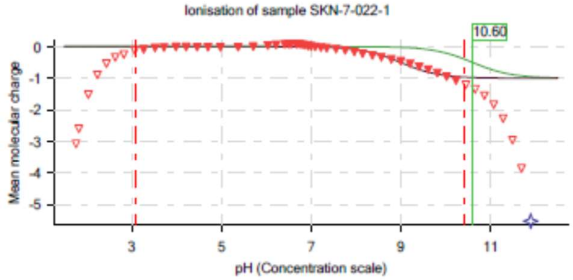

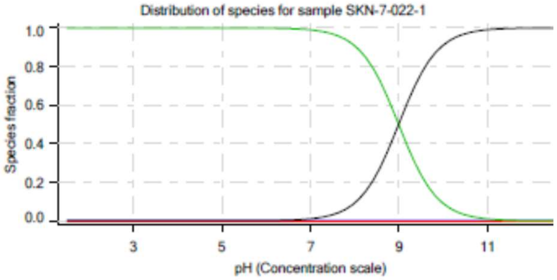

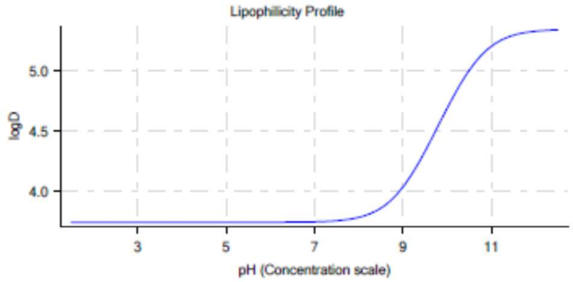

Sample logD and percent species

pH	SKN-7-022-1 logD	SKN-7-022-1 SKN-7-022-1H	SKN-7-022-1 SKN-7-022-1	SKN-7-022-1 SKN-7-022-1H*	SKN-7-022-1 SKN-7-022-1*	Comment
1.000	3.74	0.07 %	0.00 %	99.93 %	0.00 %	
1.200	3.74	0.07 %	0.00 %	99.93 %	0.00 %	Stomach pH
2.000	3.74	0.07 %	0.00 %	99.93 %	0.00 %	
3.000	3.74	0.07 %	0.00 %	99.93 %	0.00 %	
4.000	3.74	0.07 %	0.00 %	99.93 %	0.00 %	
5.000	3.74	0.07 %	0.00 %	99.92 %	0.01 %	

Sample name:	**SKN-7-022-1**	Experiment start time:	**9/1/2016 6:47:11 AM**
Assay name:	**pH-metric medium logP**	Analyst:	
Assay ID:	**16I-01006**	Instrument ID:	**T310022**
Filename:	**D:\data\Customer\16I-01006_SKN-7-022-1_pH-metric medium logP.t3r**		

Sample logD and percent species (continued)

pH	SKN-7-022-1 logD	SKN-7-022-1 SKN-7-022-1H	SKN-7-022-1 SKN-7-022-1	SKN-7-022-1 SKN-7-022-1H*	SKN-7-022-1 SKN-7-022-1*	Comment
6.000	3.74	0.07 %	0.00 %	99.83 %	0.10 %	
6.500	3.74	0.07 %	0.00 %	99.61 %	0.32 %	
7.000	3.74	0.07 %	0.00 %	98.92 %	1.01 %	
7.400	3.75	0.06 %	0.00 %	97.44 %	2.50 %	Blood pH
8.000	3.78	0.06 %	0.00 %	90.69 %	9.25 %	
9.000	4.03	0.03 %	0.00 %	49.48 %	50.49 %	
10.000	4.69	0.01 %	0.00 %	8.92 %	91.07 %	
11.000	5.21	0.00 %	0.00 %	0.97 %	99.03 %	
12.000	5.33	0.00 %	0.00 %	0.10 %	99.90 %	

Carbonate and acidity

Carbonate 0.467 mM
Acidity error 0.263 mM

Other graphs

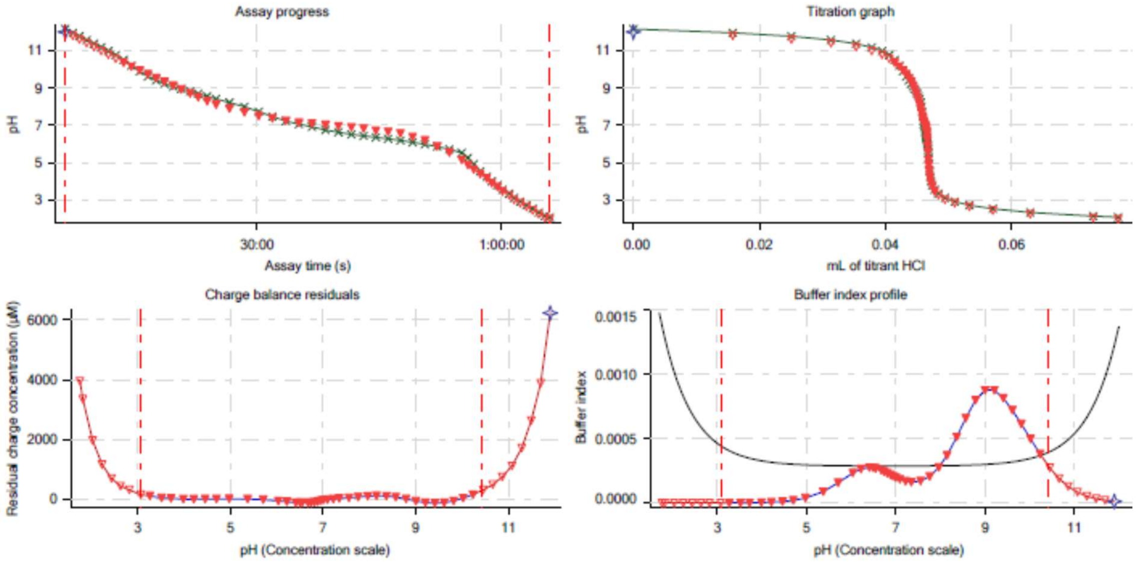

Sample name: **SKN-7-022-1**
Assay name: **pH-metric medium logP**
Assay ID: **16I-01006**
Filename: **D:\data\Customer\16I-01006_SKN-7-022-1_pH-metric medium logP.t3r**

Experiment start time: **9/1/2016 6:47:11 AM**
Analyst:
Instrument ID: **T310022**

Other graphs (continued)

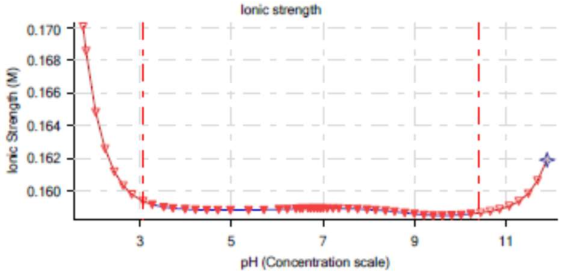

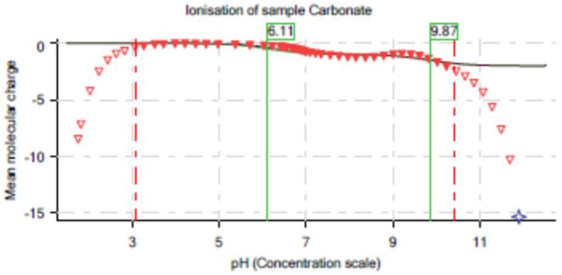

pH-metric Log P of compounds **16a**

pH-metric

Sample name:	**SKN-7-027**	Experiment start time:	**9/1/2016 11:12:29 AM**
Assay name:	**pH-metric medium logP**	Analyst:	
Assay ID:	**16I-01010**	Instrument ID:	**T310022**
Filename:	**D:\data\Customer\16I-01010_SKN-7-027_pH-metric medium logP.t3r**		

Overall results

RMSD	0.313
Average ionic strength	0.159 M
Average temperature	25.0°C
Partition ratio	0.2737 : 1
Analyte concentration range	1862.4 µM to 1969.3 µM
Total points considered	60 of 71

Warnings and errors

Errors	None
Warnings	Sample concentration factor out of range
	Excessive carbonate concentration present

Sample

SKN-7-027 concentration factor	0.622
Acid pKa 1	10.80
logP (neutral XH)	4.95
logP (X -)	7.34

Sample graphs

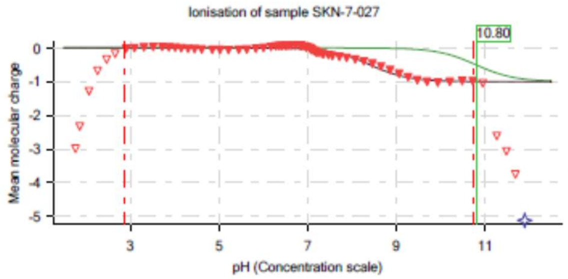

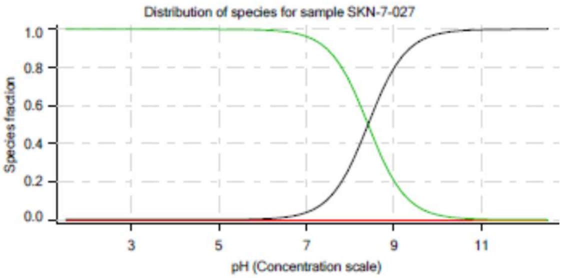

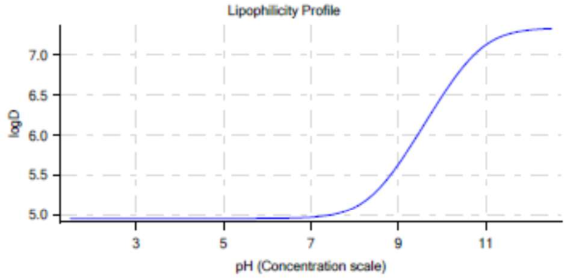

Sample logD and percent species

pH	SKN-7-027 logD	SKN-7-027 SKN-7-027H	SKN-7-027 SKN-7-027	SKN-7-027 SKN-7-027H*	SKN-7-027 SKN-7-027*	Comment
1.000	4.95	0.00 %	0.00 %	100.00 %	0.00 %	
1.200	4.95	0.00 %	0.00 %	100.00 %	0.00 %	Stomach pH
2.000	4.95	0.00 %	0.00 %	100.00 %	0.00 %	
3.000	4.95	0.00 %	0.00 %	100.00 %	0.00 %	
4.000	4.95	0.00 %	0.00 %	99.99 %	0.00 %	

Sample name:	**SKN-7-027**	Experiment start time:	**9/1/2016 11:12:29 AM**
Assay name:	**pH-metric medium logP**	Analyst:	
Assay ID:	**16I-01010**	Instrument ID:	**T310022**
Filename:	**D:\data\Customer\16I-01010_SKN-7-027_pH-metric medium logP.t3r**		

Sample logD and percent species (continued)

pH	SKN-7-027 logD	SKN-7-027 SKN-7-027H	SKN-7-027 SKN-7-027	SKN-7-027 SKN-7-027H*	SKN-7-027 SKN-7-027*	Comment
5.000	4.95	0.00 %	0.00 %	99.96 %	0.04 %	
6.000	4.95	0.00 %	0.00 %	99.61 %	0.39 %	
6.500	4.96	0.00 %	0.00 %	98.77 %	1.22 %	
7.000	4.97	0.00 %	0.00 %	96.23 %	3.76 %	
7.400	4.99	0.00 %	0.00 %	91.05 %	8.95 %	Blood pH
8.000	5.09	0.00 %	0.00 %	71.88 %	28.12 %	
9.000	5.63	0.00 %	0.00 %	20.36 %	79.64 %	
10.000	6.49	0.00 %	0.00 %	2.49 %	97.51 %	
11.000	7.13	0.00 %	0.00 %	0.26 %	99.74 %	
12.000	7.32	0.00 %	0.00 %	0.03 %	99.97 %	

Carbonate and acidity

- Carbonate 0.738 mM
- Acidity error -0.958 mM

Other graphs

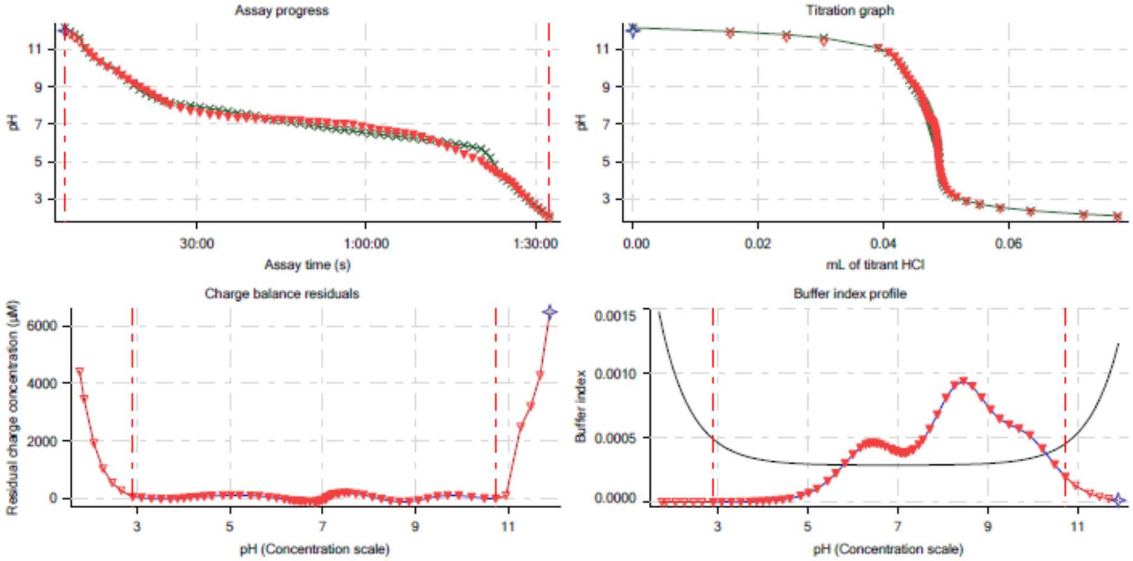

Sample name: **SKN-7-027**
Assay name: **pH-metric medium logP**
Assay ID: **16I-01010**
Filename: **D:\data\Customer\16I-01010_SKN-7-027_pH-metric medium logP.t3r**

Experiment start time: **9/1/2016 11:12:29 AM**
Analyst:
Instrument ID: **T310022**

Other graphs (continued)

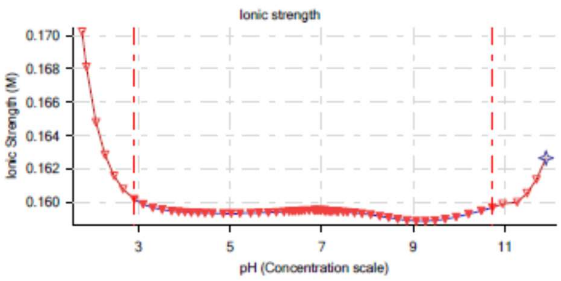

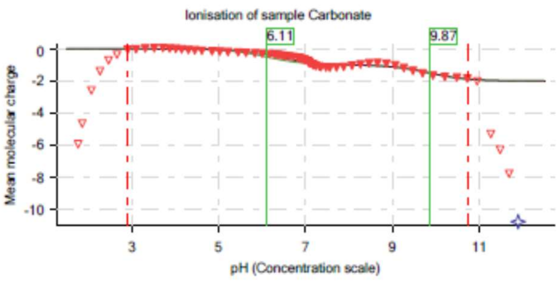

pH-metric Log P of compounds **16b**

pH-metric

Sample name:	**SKN-7-026**	Experiment start time:	**9/1/2016 10:12:57 AM**
Assay name:	**pH-metric medium logP**	Analyst:	
Assay ID:	**16I-01009**	Instrument ID:	**T310022**
Filename:	**D:\data\Customer\16I-01009_SKN-7-026_pH-metric medium logP.t3r**		

Overall results

RMSD	0.203
Average ionic strength	0.159 M
Average temperature	25.0°C
Partition ratio	0.2739 : 1
Analyte concentration range	1795.3 µM to 1900.1 µM
Total points considered	34 of 48

Warnings and errors

Errors	None
Warnings	None

Sample

SKN-7-026 concentration factor	0.732
Acid pKa 1	10.10
logP (neutral XH)	3.04
logP (X -)	3.82

Sample graphs

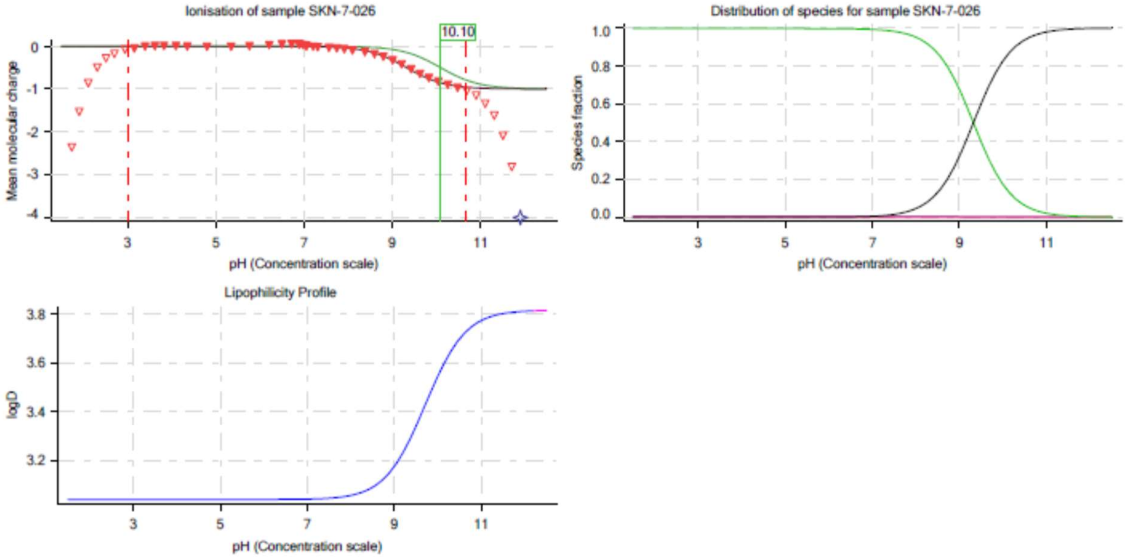

Sample logD and percent species

pH	SKN-7-026 logD	SKN-7-026 SKN-7-026H	SKN-7-026 SKN-7-026	SKN-7-026 SKN-7-026H*	SKN-7-026 SKN-7-026*	Comment
1.000	3.04	0.33 %	0.00 %	99.67 %	0.00 %	
1.200	3.04	0.33 %	0.00 %	99.67 %	0.00 %	Stomach pH
2.000	3.04	0.33 %	0.00 %	99.67 %	0.00 %	
3.000	3.04	0.33 %	0.00 %	99.67 %	0.00 %	
4.000	3.04	0.33 %	0.00 %	99.67 %	0.00 %	
5.000	3.04	0.33 %	0.00 %	99.66 %	0.00 %	

Reported at: 9/5/2016 1:45:34 PM

pH-metric

Sample name:	**SKN-7-026**	Experiment start time:	**9/1/2016 10:12:57 AM**
Assay name:	**pH-metric medium logP**	Analyst:	
Assay ID:	**16I-01009**	Instrument ID:	**T310022**
Filename:	**D:\data\Customer\16I-01009_SKN-7-026_pH-metric medium logP.t3r**		

Sample logD and percent species (continued)

pH	SKN-7-026 logD	SKN-7-026 SKN-7-026H	SKN-7-026 SKN-7-026	SKN-7-026 SKN-7-026H*	SKN-7-026 SKN-7-026*	Comment
6.000	3.04	0.33 %	0.00 %	99.62 %	0.05 %	
6.500	3.04	0.33 %	0.00 %	99.52 %	0.15 %	
7.000	3.04	0.33 %	0.00 %	99.20 %	0.47 %	
7.400	3.04	0.33 %	0.00 %	98.50 %	1.17 %	Blood pH
8.000	3.06	0.32 %	0.00 %	95.16 %	4.52 %	
9.000	3.18	0.23 %	0.02 %	67.64 %	32.11 %	
10.000	3.55	0.06 %	0.05 %	17.38 %	82.52 %	
11.000	3.77	0.01 %	0.05 %	2.06 %	97.88 %	
12.000	3.81	0.00 %	0.06 %	0.21 %	99.73 %	

Carbonate and acidity

Carbonate 0.431 mM
Acidity error 0.777 mM

Other graphs

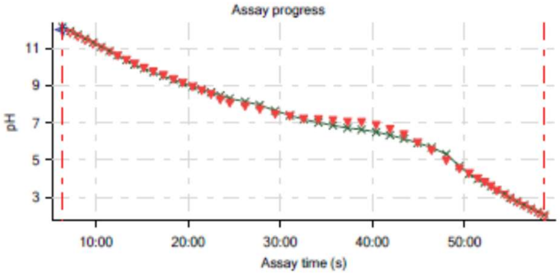

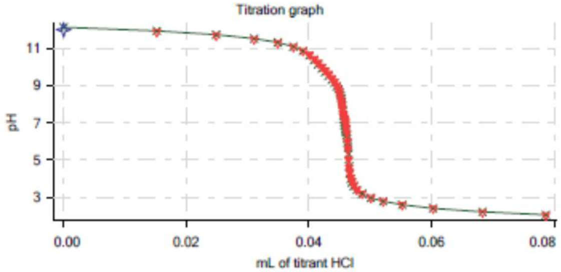

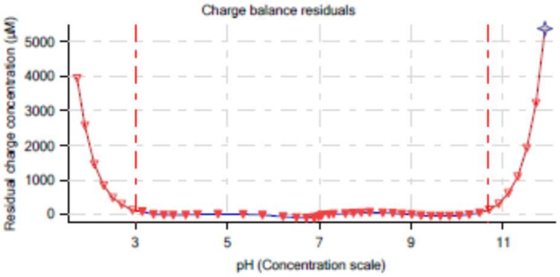

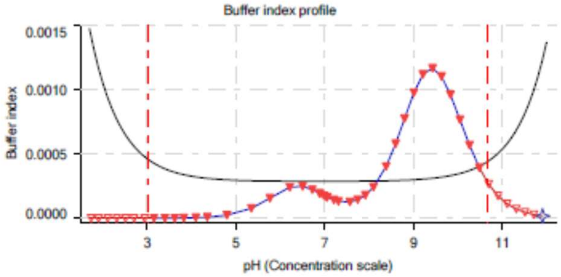

Sample name:	**SKN-7-026**	Experiment start time:	**9/1/2016 10:12:57 AM**
Assay name:	**pH-metric medium logP**	Analyst:	
Assay ID:	**16I-01009**	Instrument ID:	**T310022**
Filename:	**D:\data\Customer\16I-01009_SKN-7-026_pH-metric medium logP.t3r**		

Other graphs (continued)

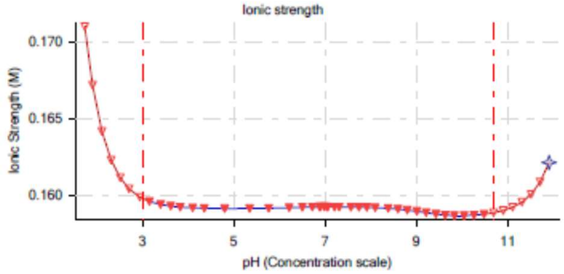

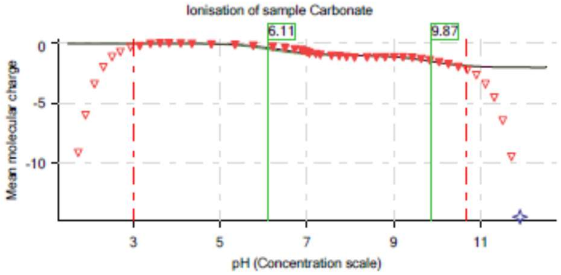

pH-metric Log P of compounds **chlorantraniliprole**

pH-metric

Sample name: **TK-1**
Assay name: **pH-metric medium logP**
Assay ID: **16H-16010**
Filename: **D:\Data\Customer\16H-16010_TK-1_pH-metric medium logP.t3r**

Experiment start time: **16/08/2016 12:55:31**
Analyst: **KRICT**
Instrument ID: **T313101**

Overall results

RMSD	0.169
Average ionic strength	0.158 M
Average temperature	25.0°C
Partition ratio	0.2765 : 1
Analyte concentration range	2039.7 µM to 2171.1 µM
Total points considered	32 of 42

Warnings and errors

Errors	None
Warnings	None

Four-Plus parameters

Alpha	0.210
S	1.0000
jH	0.1
jOH	0.1

Titrants

0.50 M HCl	0.982045	16/08/2016 12:55:31	D:\Data\Customer\16H-16006_Blank standardisation.t3r
0.50 M KOH	1.007560	16/08/2016 12:55:31	D:\Data\Customer\16G-19013_KHP_Base standardisation using KHP.t3r

Sample

TK-1 concentration factor	1.000
Acid pKa 1	10.20
logP (neutral XH)	5.25
logP (X -)	4.35

Sample graphs

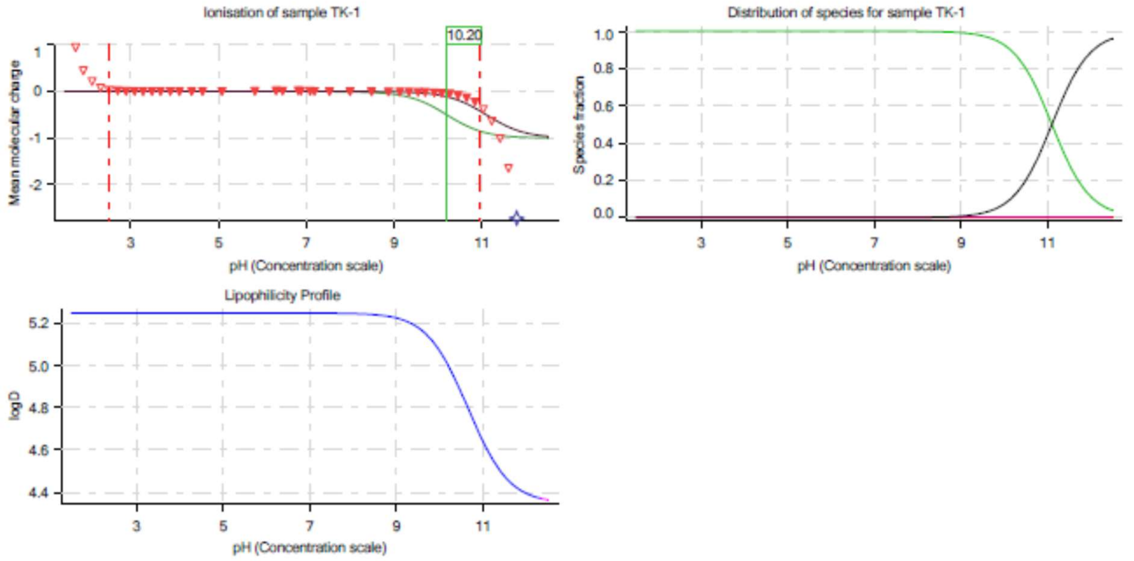

Sample name:	TK-1	Experiment start time:	16/08/2016 12:55:31
Assay name:	pH-metric medium logP	Analyst:	KRICT
Assay ID:	16H-16010	Instrument ID:	T313101
Filename:	D:\Data\Customer\16H-16010_TK-1_pH-metric medium logP.t3r		

Sample logD and percent species

pH	TK-1 logD	TK-1 TK-1H	TK-1 TK-1	TK-1 TK-1H*	TK-1 TK-1*	Comment
1.000	5.25	0.00 %	0.00 %	100.00 %	0.00 %	
1.200	5.25	0.00 %	0.00 %	100.00 %	0.00 %	Stomach pH
2.000	5.25	0.00 %	0.00 %	100.00 %	0.00 %	
3.000	5.25	0.00 %	0.00 %	100.00 %	0.00 %	
4.000	5.25	0.00 %	0.00 %	100.00 %	0.00 %	
5.000	5.25	0.00 %	0.00 %	100.00 %	0.00 %	
6.000	5.25	0.00 %	0.00 %	100.00 %	0.00 %	
6.500	5.25	0.00 %	0.00 %	100.00 %	0.00 %	
7.000	5.25	0.00 %	0.00 %	99.99 %	0.01 %	
7.400	5.25	0.00 %	0.00 %	99.98 %	0.02 %	Blood pH
8.000	5.25	0.00 %	0.00 %	99.92 %	0.08 %	
9.000	5.23	0.00 %	0.00 %	99.21 %	0.79 %	
10.000	5.07	0.00 %	0.00 %	92.64 %	7.36 %	
11.000	4.64	0.00 %	0.01 %	55.73 %	44.27 %	
12.000	4.39	0.00 %	0.01 %	11.18 %	88.81 %	

Carbonate and acidity

Carbonate 0.182 mM
Acidity error 0.680 mM

Other graphs

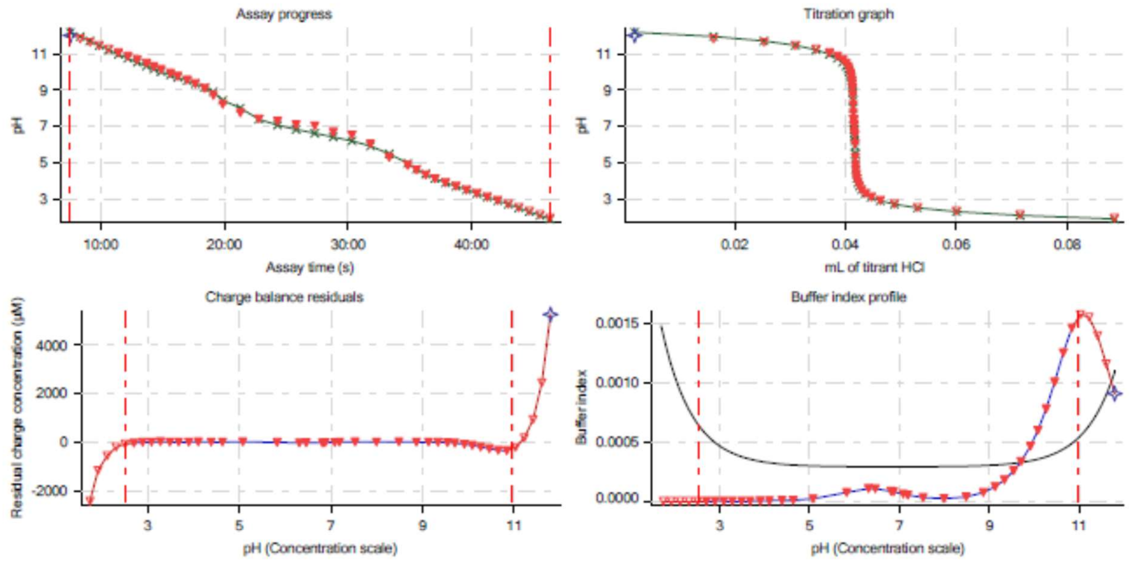

Sample name:	**TK-1**	Experiment start time:	**16/08/2016 12:55:31**
Assay name:	**pH-metric medium logP**	Analyst:	**KRICT**
Assay ID:	**16H-16010**	Instrument ID:	**T313101**
Filename:	**D:\Data\Customer\16H-16010_TK-1_pH-metric medium logP.t3r**		

Other graphs (continued)

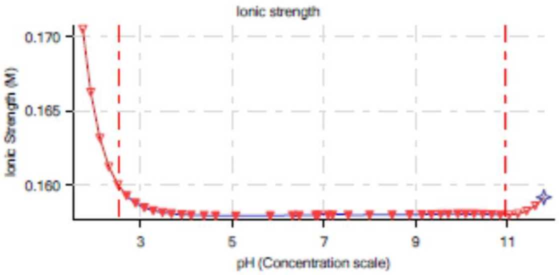

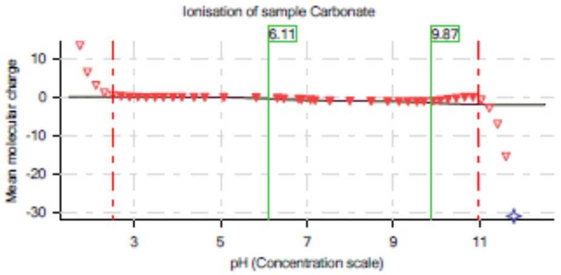

pH-metric Log P of compounds **cyantraniliprole**

pH-metric

Sample name:	**SJP-TK-02**	Experiment start time:	**9/1/2016 3:06:50 AM**
Assay name:	**pH-metric medium logP**	Analyst:	
Assay ID:	**16I-01003**	Instrument ID:	**T310022**
Filename:	**D:\data\Customer\16I-01003_SJP-TK-02_pH-metric medium logP.t3r**		

Overall results

RMSD	0.360
Average ionic strength	0.160 M
Average temperature	25.0°C
Partition ratio	0.2713 : 1
Analyte concentration range	2276.1 µM to 2416.9 µM
Total points considered	39 of 51

Warnings and errors

Errors	None
Warnings	None

Sample

SJP-TK-02 concentration factor	1.292
Acid pKa 1	10.20
logP (neutral XH)	3.50
logP (X -)	3.37

Sample graphs

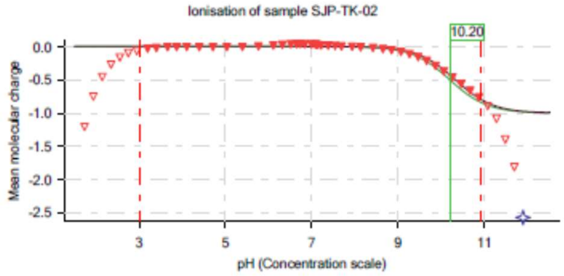

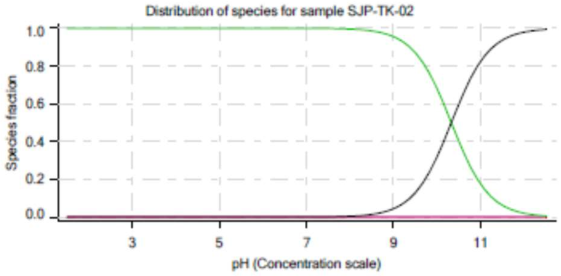

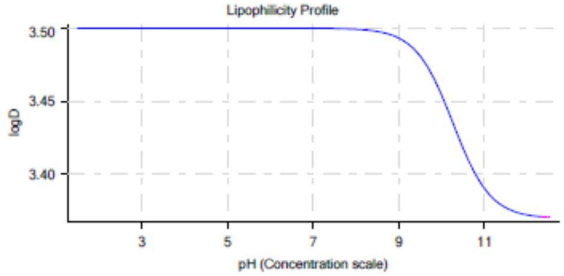

Sample logD and percent species

pH	SJP-TK-02 logD	SJP-TK-02 SJP-TK-02H	SJP-TK-02 SJP-TK-02	SJP-TK-02 SJP-TK-02H*	SJP-TK-02 SJP-TK-02*	Comment
1.000	3.50	0.12 %	0.00 %	99.88 %	0.00 %	
1.200	3.50	0.12 %	0.00 %	99.88 %	0.00 %	Stomach pH
2.000	3.50	0.12 %	0.00 %	99.88 %	0.00 %	
3.000	3.50	0.12 %	0.00 %	99.88 %	0.00 %	
4.000	3.50	0.12 %	0.00 %	99.88 %	0.00 %	
5.000	3.50	0.12 %	0.00 %	99.88 %	0.00 %	

Sample name:	**SJP-TK-02**	Experiment start time:	**9/1/2016 3:06:50 AM**
Assay name:	**pH-metric medium logP**	Analyst:	
Assay ID:	**16I-01003**	Instrument ID:	**T310022**
Filename:	**D:\data\Customer\16I-01003_SJP-TK-02_pH-metric medium logP.t3r**		

Sample logD and percent species (continued)

pH	SJP-TK-02 logD	SJP-TK-02 SJP-TK-02H	SJP-TK-02 SJP-TK-02	SJP-TK-02 SJP-TK-02H*	SJP-TK-02 SJP-TK-02*	Comment
6.000	3.50	0.12 %	0.00 %	99.88 %	0.00 %	
6.500	3.50	0.12 %	0.00 %	99.87 %	0.01 %	
7.000	3.50	0.12 %	0.00 %	99.84 %	0.05 %	
7.400	3.50	0.12 %	0.00 %	99.77 %	0.12 %	Blood pH
8.000	3.50	0.12 %	0.00 %	99.42 %	0.47 %	
9.000	3.49	0.11 %	0.01 %	95.42 %	4.46 %	
10.000	3.45	0.08 %	0.05 %	68.04 %	31.83 %	
11.000	3.39	0.02 %	0.13 %	17.59 %	82.26 %	
12.000	3.37	0.00 %	0.15 %	2.09 %	97.75 %	

Carbonate and acidity

- Carbonate 0.481 mM
- Acidity error 0.422 mM

Other graphs

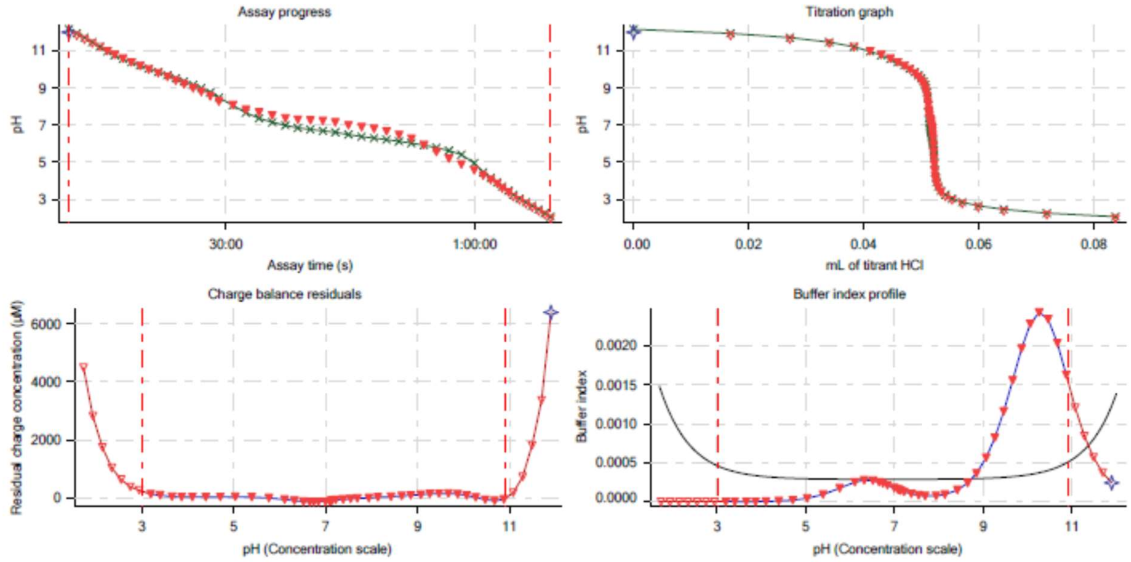

Sample name: **SJP-TK-02**
Assay name: **pH-metric medium logP**
Assay ID: **16I-01003**
Filename: **D:\data\Customer\16I-01003_SJP-TK-02_pH-metric medium logP.t3r**

Experiment start time: **9/1/2016 3:06:50 AM**
Analyst:
Instrument ID: **T310022**

Other graphs (continued)

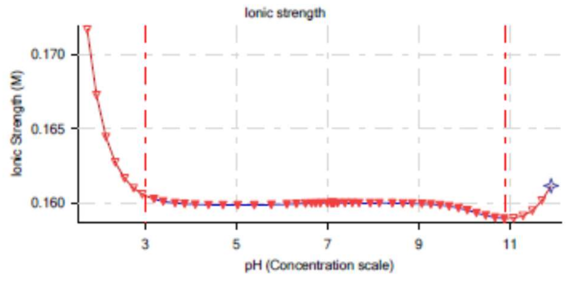

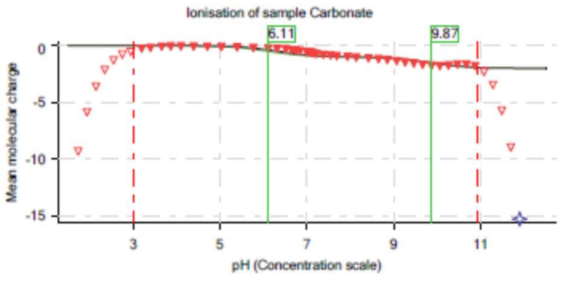

References

1. (a) S. R. Katamreddy, A. J. Carpenter, C. E. Ammala, E. E. Boros, R. L. Brashear, C. P. Briscoe, S. R. Bullard, R. D. Caldwell, C. R. Conlee, D. K. Croom, S. M. Hart, D. O. Heyer, P. R. Johnson, J. A. Kashatus, D. J. Minick, G. E. Peckham, S. A. Ross, S. G. Roller, V. A. Samano, H. R. Sauls, S. M. Tadepalli, J. B. Thompson, Y. Xu, J. M. Way, *J. Med. Chem.* **2012**, *55*, 10972; (b) J. D. Moseley, E. K. Woodman, *Org. Process Res. Dev.*, **2008**, *12*, 967; (c) Positive allosteric modulators of the nicotinic acetylcholine receptor, D. W. Piotrowski, B. N. Rogers, W. W. Mcwhorter, D. P. Walker, J. W. Corbett, V. E. Groppi, D. G. Rudmann, (Pharmacia & Upjohn Company), WO/2003/093250 A2, 2003.

2. (a) Y. Xu, T. Cong, P. Liu, P. Sun, *Org. Biomol, Chem.*, **2015**, *13*, 9742; (b) P. F. Ranken, B. G. McKinnie, *J. Org. Chem.* **1989**, *54*, 2985.

3. Preparation of aromatic thioethers as intermediates for herbicides, H. Nakagawa, M. Yamaguchi, H. Adachi, JP/2000/319251 A, 2000; (b) 3-thio or amino substituted-benzo[*b*]thiophene-2-carboxamides and 3-oxygen, thio, or amino substituted-benzofuran-2-carboxamides as inhibitors of cell adhesion, D. H. Boschelli, D. T. Connor, J. B. Kramer, P. C. Unangst, US/5350748 A, 1994.

4. *N*-(arylalkyl)-*N'*-pyrazolyl-urea, thiourea, guanidine and cyanoguanidine compounds as trka kinase inhibitors, J. F. Blake, B. J. Brandhuber, J. Haas, B. Newhouse, A. A. Thomas, S. L. Winski, (Array Biopharma Inc.), WO/2014/078331 A1, 2014.

5. A. Guilbault, B. Basdevant, V. Wanie, C. Y. Legault, *J. Org. Chem.*, **2012**, *77*, 11283.

6. (a) S. Zhou, Z. Jia, L. Xiong, T. Yan, N Yang, G. Wu, H. Song, Z. Li, *J. Agric. Food Chem.* **2014**, *62*, 6269; (b) S. Zhou, Y. Gu, M. Liu, C. Wu, S. Zhou, Y. Zhao, Z. Jia, B. Wang, L. Xiong, N. Yang, Z. Li, *J. Agric. Food Chem.* **2014**, *62*, 11054; (d) X. Hua, W. Mao, Z. Fan, X. Ji, F. Li, G. Zong, H. Song, J. Li, L. Zhou, L. Zhou, X. Liang, G. Wang, X. Chen, *Aust. J. Chem.* **2014**, *67*, 1491.
